# On Nonlinear Regression for Trends in Split-Belt Treadmill Training

**DOI:** 10.3390/brainsci10100737

**Published:** 2020-10-14

**Authors:** Usman Rashid, Nitika Kumari, Nada Signal, Denise Taylor, Alain C. Vandal

**Affiliations:** 1 Health & Rehabilitation Research Institute, Auckland University of Technology, Auckland 1010, New Zealand; nitika.kumari@aut.ac.nz (N.K.); nada.signal@aut.ac.nz (N.S.); denise.taylor@aut.ac.nz (D.T.); 2 Centre for Chiropractic Research, New Zealand College of Chiropractic, Auckland 1060, New Zealand; 3 Department of Statistics, The University of Auckland, Auckland 1010, New Zealand; alain.vandal@auckland.ac.nz

**Keywords:** motor training, gait analysis, split-belt treadmill, step length symmetry, nonlinear regression, particle swarm optimisation (PSO), akaike’s information criterion (AIC)

## Abstract

Single and double exponential models fitted to step length symmetry series are used to evaluate the timecourse of adaptation and de-adaptation in instrumented split-belt treadmill tasks. Whilst the nonlinear regression literature has developed substantially over time, the split-belt treadmill training literature has not been fully utilising the fruits of these developments. In this research area, the current methods of model fitting and evaluation have three significant limitations: (i) optimisation algorithms that are used for model fitting require a good initial guess for regression parameters; (ii) the coefficient of determination ($R^{2}$) is used for comparing and evaluating models, yet it is considered to be an inadequate measure of fit for nonlinear regression; and, (iii) inference is based on comparison of the confidence intervals for the regression parameters that are obtained under the untested assumption that the nonlinear model has a good linear approximation. In this research, we propose a transformed set of parameters with a common language interpretation that is relevant to split-belt treadmill training for both the single and double exponential models. We propose parameter bounds for the exponential models which allow the use of particle swarm optimisation for model fitting without an initial guess for the regression parameters. For model evaluation and comparison, we propose the use of residual plots and Akaike’s information criterion (AIC). A method for obtaining confidence intervals that does not require the assumption of a good linear approximation is also suggested. A set of MATLAB (MathWorks, Inc., Natick, MA, USA) functions developed in order to apply these methods are also presented. Single and double exponential models are fitted to both the group-averaged and participant step length symmetry series in an experimental dataset generating new insights into split-belt treadmill training. The proposed methods may be useful for research involving analysis of gait symmetry with instrumented split-belt treadmills. Moreover, the demonstration of the suggested statistical methods on an experimental dataset may help the uptake of these methods by a wider community of researchers that are interested in timecourse of motor training.

## 1. Introduction

Split-belt treadmill consists of two separate belts allowing for each leg to move at a different speed [1]. The split-belt can be used in two speed configurations. First, same speed configuration (SS) in which both the belts move at the same speed. Second, differential speed configuration (DS), in which the two belts move at different speeds [2]. Split-belt treadmill walking, where one leg is forced to move at a faster speed, is a well studied task in humans, animals, and using robots [3,4,5,6,7,8,9,10,11]. Motor training in response to the novelty and perturbation imposed by the split-belt treadmill walking has generated useful insights into the biomechanics and neurophysiology of gait in both healthy and pathological populations [12,13,14,15,16,17,18,19]. Using these insights, researchers have proposed a variety of gait rehabilitation paradigms for patients with motor impairments [20,21,22].

Force plates which are embedded in the belts, three-dimensional (3-D) motion capture camera system, accelerometers, and gyroscopes are generally used to record different aspects of the gait during split-belt treadmill tasks [23,24,25,26,27,28,29,30]. Step length symmetry series, which is a kinematic measure of difference between the left and the right step lengths over successive strides, is used to study motor adaptation and de-adaptation during motor training of individuals walking on the split-belt treadmill [30]. Motor adaptation refers to trial-and-error adjustments (adaptation) made in response to perturbations to well-learnt motor tasks. These adjustments return to baseline levels (de-adaptation) upon the removal of the perturbation [31]. Single and double exponential models are fitted to the step symmetry series to quantify the timecourse of adaptation and de-adaptation [27,30,32,33,34]. The exponential trends in symmetry series are in agreement with the findings of a large comparative study which included 40 datasets representing 7910 “learning series” from 24 different experiments involving tasks such as memory search, counting, mental arithmetic and motor training among others. This study concluded that the exponential law was a better representation of the learning trends than the power law [35]. However, there are three important limitations of the nonlinear regression methods used to fit and evaluate the exponential models in split-belt treadmill literature.

First, the algorithms that are used to fit the nonlinear models are not explicitly stated [30,33,34,36,37,38,39,40,41,42,43,44,45,46]. We were able to find one article in which the authors had explicitly stated that they used the Levenberg–Marquardt algorithm [47]. The results of this algorithm and other algorithms that are based on gradient decent are sensitive to the initial parameter guesses [48,49]. This is due to the fact that, unlike linear regression problems that have a single minimum in the solution space of parameters, nonlinear regression has multiple minima in the solution space and these algorithms follow the gradient to converge to the nearest local minimum starting at the initial guess for parameters. The most commonly reported softwares for fitting these models such as MATLAB’s Curve Fitting Toolbox (MathWorks, Inc., Natick, MA, USA), CoStat [50] and SigmaPlot [51] use these algorithms by default although better alternatives are available in these softwares. The initial guesses need to be varied across individuals, sessions, and groups. This reduces the reproducibility of results across studies as reporting of initial guesses is not useful. Second, the choice of the particular function (single or double exponential) to represent the symmetry series is usually justified by citing earlier research or by evaluation using the coefficient of determination ($R^{2}$), which is interpreted as a measure of the goodness of the fit. $R^{2}$ is considered to be an inadequate measure for nonlinear models, such as models based on single and double exponential functions [52]. Third, the parameters of the models fitted to data are compared while using confidence intervals and the methods used for obtaining the confidence intervals are not explicitly stated. Some of these methods result in confidence intervals, which can be “extremely misleading” [53,54]. This, in turn, may compromise the inferences drawn.

The aim of this research is to address these three limitations by adapting methods for split-belt treadmill training from established nonlinear regression literature [55,56,57]. We propose a transformed set of parameters with a common language interpretation that is relevant to split-belt treadmill training for both the single and double exponential models. We propose parameter bounds for the exponential models which allow the use of particle swarm optimisation for model fitting without an initial guess for the regression parameters [58]. For model evaluation and comparison, we propose the use of residual plots and Akaike’s information criterion (AIC) [59]. A method for obtaining confidence intervals that does not require the assumption of a good linear approximation is also suggested [60]. A set of MATLAB (MathWorks, Inc., Natick, MA, USA) functions developed to apply these methods are also presented. Using these functions, the application of the proposed methods is illustrated on an experimental dataset for both the group-averaged and participant symmetry series. To the best of our knowledge, the key novel aspects of this research are: (i) a transformed set of parameters with a common language interpretation for the exponential models used in split-belt treadmill training research, (ii) parameter bounds for the regression parameters to allow for nonlinear regression using particle swarm optimisation without providing initial guess, (iii) application of existing statistical methods such as AIC and confidence intervals without the linearity assumption to split-belt treadmill training, and (iv) providing publicly available software for application of the aforementioned methods.

## 2. Materials and Methods

### 2.1. Experimental Dataset

The experimental dataset is introduced first to facilitate the reading of the concepts introduced later in the report. The dataset is taken from a randomised controlled trial involving healthy participants walking on an instrumented split-belt treadmill (Bertec Corporation, Columbus, OH, USA). The trial investigated the effect of cerebellar transcranial direct current stimulation (ctDCS) on motor learning. Ethics approval (16/338) for the study was obtained from Auckland University of Technology Ethics Committee (AUTEC). Each participant attended four sessions; three intervention sessions on consecutive days and a one week apart follow-up session. During the intervention sessions, the participants received either active ctDCS or sham ctDCS during split-belt treadmill walking. At follow-up, split-belt treadmill walking was undertaken without ctDCS. The participants were instructed to stand in the middle of the treadmill with one foot on each belt, hold onto a front rail, and looking straight ahead whilst walking. The participant’s fastest comfortable walking speed was taken as the average from three trials where it was assessed by slowly increasing treadmill speed until the participant reported an inability to comfortably tolerate any further increase. The speed of the slower belt was set to half that of the fast belt. During baseline, participants walked with symmetrical belts at the slow gait speed. After 2 min. of walking, the treadmill was stopped, and the ctDCS unit was turned on. During the adaptation phase, the treadmill was restarted at the slow gait speed, and then the belt speed of the fast belt was increased until the fast gait speed was attained. This asymmetrical speed ratio of 2:1 was then maintained for 15 min. Finally, in the de-adaptation phase, the ctDCS was turned off, and the participant walked for 10 min. with both belts symmetrical at the slow speed. A motion capture system (Vicon Nexus 2.4, Vicon Motion Systems Ltd., Los Angeles, Culver City, CA, USA) was used to record force and position data from treadmill force plates and reflective markers, respectively. The position of reflective markers was captured via nine-cameras at a frame rate of 200 Hz. The data were recorded during the last minute of the baseline phase and throughout the adaptation and the de-adaptation phase [61]. A MATLAB-based implementation of the Detection and Correction Algorithm (DACA) was utilised for determining the gait events: heel-strikes and toe-offs for each foot [62]. Fast step length and slow step length were calculated once the gait events were detected.

**Figure 1:**
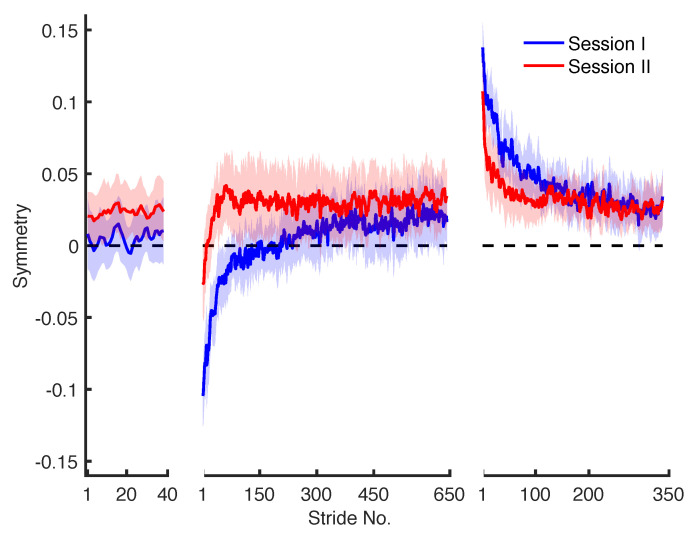
Group-averaged (*n* = 15) step length symmetry series with 95% confidence intervals (*z* = 1.96) for baseline, adaptation and de-adaptation phases from two consecutive days of training. A 3-point moving average filter was used to smooth the data for aesthetic considerations.

To evaluate the proposed methods, data from the first and the second session of the control group (sham ctDCS, *n* = 15) from the trial are used in this report. The motor performance of the participants in the two sessions is quantified while using step length symmetry. This is a unitless variable calculated for each stride as the difference in fast and slow step lengths, and normalised to their sum to cater to inter-individual variability in step lengths. The step symmetry (*y*) for the *n*th stride is calculated as follows [30]:

(1) $$y\left(n\right)=\frac{l_{f}\left(n\right)-l_{s}\left(n\right)}{l_{f}\left(n\right)+l_{s}\left(n\right)},$$

where $l_{f}$ and $l_{s}$ are the step lengths that correspond to the fast and the slow leg. Perfect symmetry between fast leg and slow leg is indicated by a symmetry value of zero, whereas a positive value indicates a longer step length of the fast leg and a negative value indicates a longer step length of the slow leg. The step length symmetry during baseline, adaptation, and de-adaptation phases for the two sessions is plotted in Figure 1.

### 2.2. Exponential Models

#### 2.2.1. Single Exponential Model

**Figure 2:**
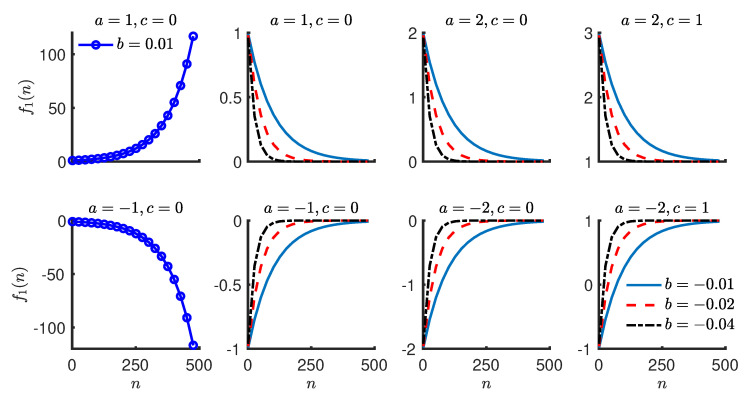
Single exponential function with different parameter values. The first two plots in the first column show growing exponential functions. The remaining plots show decaying exponential functions. *c* determines the final value of the function. *a* determines the change in the exponential function from the beginning to the end. *b* determines the relative rate of change.

A single exponential model fitted to step length symmetry series is given in the following equation:

(2) $$y\left(n\right)=f_{1}\left(n;\theta \right)+\epsilon \left(n\right),\mathrm{where}f_{1}\left(n;\theta \right)=a\times e^{bn}+c,$$

where y(n) is the symmetry value for the *n*th stride, θ=[a,b,c] are the parameters of the exponential model, and $\epsilon \left(n\right)∼N\left(0,\sigma_{\epsilon }^{2}\right)$ is a zero-mean Gaussian process with variance $\sigma_{\epsilon }^{2}$ at the *n*th stride. ϵ(n) represents the residual in the symmetry value for the *n*th stride, which is not explained by the exponential function $f_{1}\left(n\right)$. One interpretation of the exponential model is that it represents the systematic trend in a symmetry series with an exponential function and the random variability with a zero-mean Gaussian process. The random variability is attributed to biological variability and measurement error. The exponential function changes at a rate proportional to its current value. Figure 2 shows exponential functions with different parameter values. The two functions in the first column have b>0 and are growing exponentially in positive and negative directions. The growing exponentials are not of much use in fitting a model to the step length symmetry series as they do not converge to a finite value. For example, if the exponential function in the top left corner of Figure 2 is used to model step length symmetry, it implies an ever increasing symmetry value over successive strides. All of the remaining exponentials are decaying and they have one thing in common, which is that b<0. Under this condition, the parameters of the exponential function have intuitive interpretations for the step length symmetry series.

The value of *c* represents the symmetry at the end of training. The value of *a* represents the change in symmetry value from start to end of the training phase. This can be observed in Figure 2, where the amount of change *a* does not differ across exponentials with same *a* under variation in other parameters. The sign of *a* represents the direction from which the symmetry series approaches its final value (*c*). The rate of change of an exponential function is proportional to its current value and *b* is the constant in this proportionality relationship. A function with larger absolute value of *b* approaches *c* at a relatively faster rate when compared to an exponential function with a smaller absolute value of *b*. Yet another interpretation of the value of *b* is that $\left\lfloor \frac{\ln(2)}{\left|b\right|}\right\rfloor$ represent the expected number of strides required to complete approximately 50% of the total change (*a*). ln denotes the natural logarithm such that $\ln(e^{x})=x$, where *x* is any real number. |x| is the absolute value of a real number *x*. $\left\lfloor x\right\rfloor$ is the largest integer less than or equal to the real number *x*. For example, $\left\lfloor 3.14\right\rfloor$ evaluates to 3. The standard deviation ($\sigma_{\epsilon }$) of the residuals (ϵ(n)) also has an intuitive interpretation. As the residuals are assumed to be normal (zero-mean Gaussian), one standard deviation ($\sigma_{\epsilon }$) accounts for approximately 68% of the residuals. A simpler interpretation is that approximately 68% of the values in a symmetry series fall within a distance of one standard deviation ($\sigma_{\epsilon }$) on either side of the exponential trend.

##### Common Language Interpretation

In summary, a single exponential model for step length symmetry series implies that the step lengths get symmetrical over time and the rate of change of symmetry becomes smaller over successive strides. The parameters of the model represent the initial asymmetry (a+c), change in symmetry (*a*) from beginning to the end of the training phase, the number of strides ($\left\lfloor \frac{\ln(2)}{\left|b\right|}\right\rfloor$) taken to complete approximately 50% of the change, the asymmetry (*c*) at the end of the training phase, and the distance ($\sigma_{\epsilon }$) from the exponential trend, which accounts for approximately 68% of the symmetry values.

#### 2.2.2. Double Exponential Model

**Figure 3:**
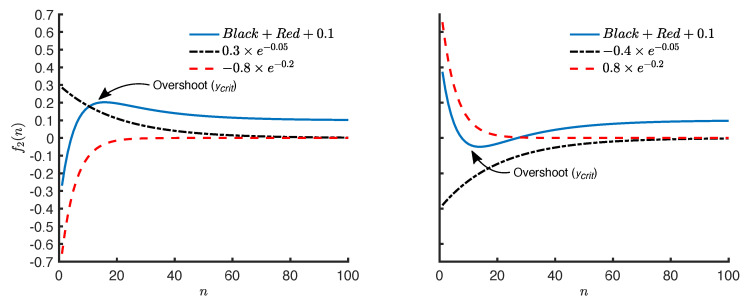
Double exponential functions with an overshoot that is produced by a positive and a negative exponential trend.

A double exponential model is given in the following equation:

(3) $$y\left(n\right)=f_{2}\left(n;\theta \right)+\epsilon \left(n\right),\mathrm{where}f_{2}\left(n\right)=a_{s}\times e^{b_{s}n}+a_{f}\times e^{b_{f}n}+c,$$

where y(n) is the symmetry value for the *n*th stride, $\theta =[a_{s},\;b_{s},\;a_{f},\;b_{f},\;c]$ are the parameters of the exponential model, and $\epsilon \left(n\right)∼N\left(0,\sigma_{\epsilon }^{2}\right)$ is a zero-mean Gaussian process with variance $\sigma_{\epsilon }^{2}$ at the *n*th stride. ϵ(n) represents the residual in the symmetry value for the *n*th stride that is not explained by the exponential function $f_{2}\left(n\right)$. The double exponential model represents two concurrent exponential changes $a_{s}$ and $a_{f}$ occurring at slow ($b_{s}$) and fast ($b_{f}$) rates, respectively. The intuitive interpretation of the single exponential model also applies to the double exponential model. However, it differs from the single exponential model, as it decomposes the systematic trend in a symmetry series into two underlying exponential trends. One of the trends changes at a relatively slower rate compared to the other trend. This implies that the faster trend vanishes in a fewer number of strides compared to the slower trend. It also implies that, across the successive strides, the systematic trend in the symmetry series increasingly resembles the slower trend. An additional property of the double exponential model is its ability to represent an overshoot in a symmetry series by decomposing the series into a positive and a negative exponential trend. An overshoot occurs when the symmetry series swings in the opposite direction of its origin. Figure 3 shows two examples.

The value of overshoot for the double exponential model is given by the following equation:

(4) $$y_{crit}\left(\theta \right)=a_{s}\times e^{b_{s}n_{crit}}+a_{f}\times e^{b_{f}n_{crit}}+c,\mathrm{where}n_{crit}=\frac{\ln\left[-\frac{a_{s}b_{s}}{a_{f}b_{f}}\right]}{b_{f}-b_{s}}$$

This equation is derived by setting *n* equal to the critical point in $f_{2}\left(n\right)$ where the first-order derivative of $f_{2}$ with respect to *n* is 0. An analysis of Equation (4) suggests that an overshoot only occurs when $a_{s}$ and $a_{f}$ have opposite signs. This is an implication of the the fact that both $b_{s}$, $b_{f}$ are negative and ln is only defined for positive numbers, thus, requiring $\left[-\frac{a_{s}b_{s}}{a_{f}b_{f}}\right]$ to be positive with opposite signs for $a_{s}$, $a_{f}$.

##### Common Language Interpretation

In summary, the parameters of the double exponential model represent the initial asymmetry ($a_{s}+a_{f}+c$), change in symmetry ($a_{s}+a_{f}$) from beginning to the end of the training phase, the number of strides ($\left\lfloor \frac{\ln(2)}{|b_{s}|}\right\rfloor$, $\left\lfloor \frac{\ln(2)}{|b_{f}|}\right\rfloor$) taken to complete approximately 50% of the changes, the asymmetry (*c*) at the end of the training phase, the overshoot in the exponential trend ($y_{crit}$), and the distance ($\sigma_{\epsilon }$) from the exponential trend, which accounts for approximately 68% of the symmetry values.

### 2.3. Model Fitting

#### 2.3.1. Parameter Bounds

An important challenge for fitting the exponential models is the specification of the parameter bounds. In the following paragraphs we argue for selection of particular bounds for each parameter of the exponential models under the assumption that the symmetry value varies within [−1, 1].

##### Bounds for *c*

As *c* represents the symmetry value at the end of the series, its maximum value can not exceed 1 or −1. In most practical situations, the value of *c* is expected to remain close to zero. Nevertheless, for the sake of generality, the bounds for *c* are specified as [−1, 1].

##### Bounds for *b*, $b_{s}$, $b_{f}$

Because it has been argued earlier that the positive values for *b*, $b_{s}$ or $b_{f}$ result in exponentially growing functions, therefore, only negative values are admissible. For approximately 50% change to occur in one stride, which is an impractical case, the largest absolute value for *b*, $b_{s}$, or $b_{f}$ would be ln(2). Using these two arguments, the lower and upper bounds for *b*, $b_{s}$, and $b_{f}$ are set at −ln(2) and 0, respectively.

##### Bounds for *a*, $a_{s}$, $a_{f}$

**Figure 4:**
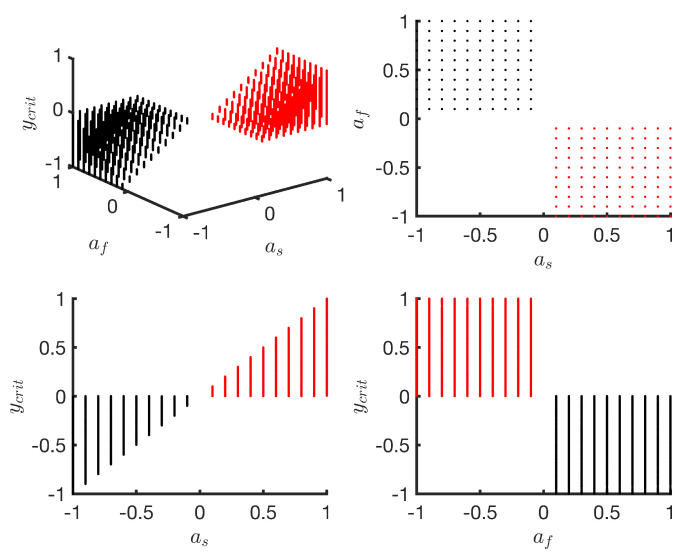
Numerical solutions for overshoot value ($y_{crit}$) for the double exponential model with respect to parameters $a_{s}$, $a_{f}$.

As *a* represents the change in symmetry value from start to end of the training phase, the maximum change cannot be greater than 2. Thus, the lower and upper bounds for *a* are set at −2 and 2, respectively. Setting the bounds for $a_{s}$, $a_{f}$ turns out to be a non-trivial problem and requires further insights. The total change in symmetry from start to the end of the series in a double exponential model is represented by $a_{s}+a_{f}$. Thus, the value of $a_{s}+a_{f}$ is constrained to remain within [−2, 2]. When $a_{s}$, $a_{f}$ are both positive or both negative, any value for total change ($a_{s}+a_{f}$) from the closed set [−2, 2] can be achieved by assigning $a_{s}$, $a_{f}$ from the set [−1, 1]. However, when $a_{s}$, $a_{f}$ have opposite signs, as it is the case in Figure 3, which presents examples of overshoot, an analysis of the relationship between the value of the overshoot and the values of $a_{s}$, $a_{f}$ is needed. Further insights into the relationship between $a_{s}$, $a_{f}$ and $y_{crit}$ are obtained with numerical solutions of Equation (4) by sampling $a_{s}$, $a_{f}$ from the set [−1, 1] with a step of 0.1; $b_{s}$, $b_{f}$ from the set [−ln(2), −10^−3^] and setting *c* to 0. Additional constraints, such as $b_{f}$ < $b_{s}$ and $a_{s}+a_{f}$ ≤ 2, are also applied. Figure 4 shows the results.

This analysis suggests that any value for the overshoot $y_{crit}$ from the set [−1, 1] can be achieved by assigning $a_{s}$ and $a_{f}$ from the set [−1, 1] excluding 0. Moreover, for a positive overshoot in the set (0, 1], $a_{s}$ has to be a positive number from the set (0, 1] and $a_{f}$ has to be negative from the set [−1, 0). Similarly, for a negative overshoot from the set [−1, 0), $a_{s}$ has to be a negative number from the set [−1, 0) and $a_{f}$ has to be a positive number from the set (0, 1]. Although some of these sets are open, they are treated as closed sets in the subsequent sections and, if the parameter selection algorithm converges to a boundary case where one or more of the parameters are 0, it is interpreted as a double exponential model without an overshoot or a degenerate case that is no better than a single exponential model. The bounds for $a_{s}$, $a_{f}$ can be further narrowed by determining the direction from which the symmetry series approaches its final value. We infer this direction from the sign of the difference in the mean symmetry of first 50 strides and the last 50 strides. This is not guaranteed to work in all cases and caution is warranted. For example, when the symmetry series approaches its final value from a positive direction, it implies that the total change from start to the end of the training phase is positive. Table 1 summarises the parameter bounds.

#### 2.3.2. Optimisation Problems

The parameters of the exponential models can be estimated by solving nonlinear least-squares optimisation problems [60]. For the single exponential model, the optimisation problem is stated, as follows:

(5) $$\begin{array}{cccc} \; & \min_{\theta } & \; & \sum_{n=1}^{N}{\left[y\left(n\right)-f_{1}\left(n;\theta \right)\right]}^{2}\\ \; & \; & & \theta^{l}\leq \theta \leq \theta^{u} \end{array}$$

This optimisation problem requires a set of parameters θ for the exponential function $f_{1}\left(n;\theta \right)$, such that the sum of squared errors ($\sum_{n=1}^{N}{\left[y\left(n\right)-f_{1}\left(n;\theta \right)\right]}^{2}$) is minimised. Additionally, the selected set of parameters should be within the lower and upper bounds specified elementwise by $\theta^{l}$ and $\theta^{u}$, respectively. For the single exponential model, the parameters are θ=[a,b,c] and their bounds are summarised in Table 1.

The double exponential model can be fitted by solving a similar optimisation problem. However, two additional constraints are required in its case. First, to make sure that $b_{s}$ always corresponds to a slower decay (a smaller negative number) than $b_{f}$, $b_{f}<b_{s}$ is required as a constraint. Second, a constraint on the total change in symmetry ($|a_{s}+a_{f}|\leq 2$) is required. Adding the first constraint to the optimisation problem is non-trivial, as it results in an open feasible set. One possible way to circumvent this problem is to reduce the strict inequality to an inequality such as $b_{f}-b_{s}+\delta \leq 0$, where δ is a small constant that determines the smallest allowed difference between $b_{f},b_{s}$. δ is set to 10^−3^ with the assumption that it is an adequately small number and it does not reduce the generality of the model in any considerable way. We added these constraints as penalty functions [63].;

(6) $$\begin{array}{cccc} \; & \min_{\theta } & \; & \lambda_{1}\max{\left(0,b_{f}-b_{s}+10^{-3}\right)}^{2}+\lambda_{2}\max(0,|a_{s}+a_{f}{\left|-2\right)}^{2}+\sum_{n=1}^{N}{\left[y\left(n\right)-f_{2}\left(n;\theta \right)\right]}^{2}\\ \; & \; & & \theta^{l}\leq \theta \leq \theta^{u}, \end{array}$$

where $\lambda_{1}$, $\lambda_{2}$ are weight parameters to penalise the search strategy if it converges towards a solution, where $b_{s}$ corresponds to a faster rate, or, where the total change in symmetry is larger than 2. The weights are both fixed at $10^{3}$ with some experimentation suggesting that this value (practically the penalty is $10^{-3}$ when the difference between $b_{s}$ and $b_{f}$ is 0 or $|a_{s}+a_{f}|$ is larger than 2 by $10^{-3}$) is not too small to be ignored by the search strategy when converging to a solution and not too large to hamstring the search strategy from exploring for suitable minima in the feasible solution set. θ is $\left[a_{s},b_{s},a_{f},b_{f},c\right]$ in this case and the bounds are specified in Table 1. As the double exponential model has two sets of bounds, depending on the presence or absence of overshoot, the optimisation is performed with both set of bounds and the solution with the smaller cost is chosen.

#### 2.3.3. Optimisation Algorithm

Methods, such as Gauss–Newton or Levenberg–Marquardt, are generally used for model fitting [48,64]. The limitation of these methods is that an initial guess for the parameters is required and this guess then dictates which local minimum from the feasible set is selected by the algorithm. To overcome this limitation, researchers have sought global approaches to optimisation, such as the particle swarm algorithm [58]. A detailed tutorial explaining the particle swarm algorithm along with practical examples can be found in [65]. The advantage of using the particle swarm algorithm over traditional gradient-based methods is that it does not require a good initial guess for the parameters that can be initialised to random values within their bounds, and its results are less sensitive to the initial guess [66,67,68,69,70]. However, optimisation is computationally expensive and it can take a longer time to converge to a solution when compared to a traditional gradient-based algorithm. To strike a balance between the global approach of the particle swarm algorithm and computational complexity, we use a large cost function tolerence ($10^{-3}$) with the particle swarm algorithm and follow it by an interior-point algorithm with cost function tolerance set at $10^{-6}$ [71]. Moreover, to reduce the likelihood of the algorithm converging to a poor local minimum, we run the same procedure twice, starting at random initial guesses for the parameters, and choose the solution that has the smaller cost.

### 2.4. Model Evaluation and Selection

As $R^{2}$ is not an appropriate measure of the goodness of fit for nonlinear regression [52], we suggest visual analysis of the model residuals in order to evaluate its adequacy in representing the data [72,73]. Qualitative statistical plots such as residuals across strides plot, histogram and QQ-plot of residuals are used. The residuals across strides plot is used to check the assumption whether the residual variance is constant across the strides or not? This assumption is made for both the single and double exponential models, as specified in Section 2.2.1 and Section 2.2.2. The violation of this assumption implies that either the model has failed to explain the systematic trend with exponential functions or the random variability has a more complex structure than a zero-mean constant variance Gaussian process. The residuals histogram and QQ-plot are used to check the assumption of normality, which is also necessary for obtaining confidence intervals and the AIC. It is worthwhile to emphasise that the histogram be interpreted with care, as it can be misleading and a greater weight be given to the QQ-plot.

We propose the use of AIC to compare the single and double exponential models fitted to the same symmetry series [59]. AIC penalises both under fitting and over fitting and the model with smaller AIC is considered superior. To apply the AIC criterion, we compute the difference between the AIC value from the double exponential model and the single exponential model (AIC(Double) − AIC(Single)) and select the double exponential model if this difference is smaller than −2 or, otherwise, select the single exponential model. AIC for the exponential models is computed while using the following formula:

(7) $$AIC=2k+N\ln\left[\sum_{n=1}^{N}\hat{\epsilon }{\left(n\right)}^{2}\right],$$

where *k* is the number of estimated parameters which is 4 for the single exponential and 6 for the double exponential model, including the estimated residual variance (${\hat{\sigma_{\epsilon }}}^{2}$). An important assumption for the use of AIC in nonlinear regression is that the residuals should be normally distributed.

### 2.5. Confidence Intervals for Parameters

95% confidence intervals for the parameters of the exponential models are used for inference. No overlap across two confidence intervals is interpreted as statistically significant difference between the estimated parameters [74].

#### 2.5.1. Based on Linearisation

The least computationally expensive method for constructing confidence intervals of individual parameters of the single and double exponential models exploits the linearisation of the models [75]. The (1−α)% confidence interval for the least-squares estimate of the *j*th parameter (${\hat{\theta }}_{j}$) is given by the following equation:

(8) $$|\theta_{j}-{\hat{\theta }}_{j}|\leq {{\hat{V}}_{jj}}^{1/2}t_{N-p,1-\alpha /2},$$

where $t_{N-p,1-\alpha /2}$ is the 1−α/2 quantile of the *t*-distribution with N−p degrees of freedom. *p* is the number of elements of θ. ${\hat{V}}_{jj}$ is the jjth element of the estimated variance matrix ($\hat{V}$) that is given by the following equation:

(9) $$\begin{array}{c} \hat{V}=s^{2}{\left[J^{T}\left(\hat{\theta }\right)J\left(\hat{\theta }\right)\right]}^{-1}\\ s^{2}=\frac{\sum_{n=1}^{N}{\left[y\left(n\right)-f\left(n;\hat{\theta }\right)\right]}^{2}}{N-p} \end{array}$$

Whereas, $J(\hat{\theta })$ is the N×p Jacobian matrix representing a linear approximation of the exponential function at $\hat{\theta }$. For the single and the double exponential functions, the *n*th row of the respective Jacobian is given by following equations in terms of its parameters:

(10) $$\begin{array}{c} J_{n,*}\left(\left[{\hat{a}}_{1},\;{\hat{b}}_{1},\;{\hat{c}}_{1}\right]\right)=\left[e^{{\hat{b}}_{1}n}\;\;{\hat{a}}_{1}ne^{{\hat{b}}_{1}n}\;\;1\right]\\ J_{n,*}\left(\left[{\hat{a}}_{1},\;{\hat{b}}_{1},\;{\hat{a}}_{2},\;{\hat{b}}_{2},\;{\hat{c}}_{1}\right]\right)=\left[e^{{\hat{b}}_{1}n}\;\;{\hat{a}}_{1}ne^{{\hat{b}}_{1}n}\;\;e^{{\hat{b}}_{2}n}\;\;{\hat{a}}_{2}ne^{{\hat{b}}_{2}n}\;\;1\right] \end{array}$$

This is the method that is used by default to construct confidence intervals in software packages, such as MATLAB (MathWorks, Inc.) [76]. This method relies on two assumptions, (i) the normality of the residuals and (ii) the validity of the linearisation of the model.

#### 2.5.2. Without Linearisation

The (1−α)% confidence intervals of the *j*th parameter without the need to linearise the underlying model can be obtained by numerically solving the following equation for $\theta_{j}^{*}$ [60]:

(11) $$\begin{array}{c} F_{1-\alpha }^{1,N-p}=\left(N-p\right)\frac{\tilde{S}\left(\theta_{j}^{*}\right)-S\left(\hat{\theta }\right)}{S(\hat{\theta })}\\ S\left(\theta \right)=\sum_{n=1}^{N}{\left[y\left(n\right)-f\left(n;\theta \right)\right]}^{2}, \end{array}$$

where $S(\hat{\theta })$, $\tilde{S}\left(\theta_{j}^{*}\right)$ are the residual sum of squares obtained, respectively, at the least-squares parameter estimate ($\hat{\theta }$), and by fixing the *j*th parameter at $\theta_{j}^{*}$ and solving the optimisation problem for the rest of the parameters. $F_{1-\alpha }^{1,N-p}$ is the 1−α quantile of the F-distribution with 1,N−p degrees of freedom. This equation has two roots, one above and one below the estimated least-squares parameter $\hat{\theta_{j}}$. We find the roots of the above equation with MATLAB’s *fzero* function, which uses a combination of bisection, secant, and inverse quadratic interpolation methods [77]. *fzero* is set up to iteratively converge to the lower 95% confidence interval for $\hat{\theta_{j}}$ in the interval defined by the lower bound of parameter $\theta_{j}$ and its least-squares estimate $\hat{\theta_{j}}$. Similarly, *fzero* is set up to iteratively converge to the upper 95% confidence interval for $\hat{\theta_{j}}$ in the interval defined by its least-squares estimate $\hat{\theta_{j}}$ and the upper bound of parameter $\theta_{j}$. If the algorithm fails to find a sign difference at these bounds, the range is extended to $\pm 10^{2}$. Although this method is computationally expensive when compared to the linearisation method, it results in confidence intervals that are more “exact”, precisely because it does not make the linearisation assumption regarding the fitted model [60].

### 2.6. MATLAB Implementation of Methods

A MATLAB (MathWorks, Inc., Natick, MA, USA) implementation of the methods described in the above sections is available online (https://github.com/GallVp/knkTools). These functions can be used to fit exponential models, plot diagnostic information along with AIC and obtain confidence intervals. The model fitting is fully automated and the relevant function only requires a vector of symmetry series as input. Moreover, four simulated symmetry series are also provided with the implementation as examples. These symmetry series are simulated from the parameters of the models that are fitted to data shown in Figure 1.

## 3. Results

### 3.1. Group-Averaged Symmetry Series

#### 3.1.1. Model Selection

**Figure 5:**
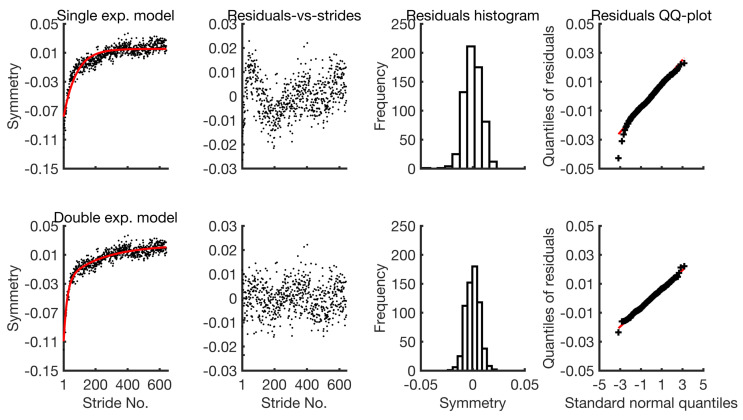
Fit and diagnostic information for the single and the double exponential models.

The fitted models along with their residuals are shown in Figure 5 for the adaptation phase from session I.

The residuals of the single exponential model clearly show its inadequacy in representing the data. The residuals-vs-strides plot shows that it violates the assumption of constant variance across strides as the dispersion of the residuals is not uniform. The histogram suggests that its residuals are slightly skewed. By contrast, the behaviour of the residuals from the double exponential model is less dependent on the stride number and their quantiles closely resemble those of the normal distribution. There is practically no skewness. Similar plots for the remaining group-averaged symmetry series are provided in the Supplementary File. The AIC values for the single and double exponential models from both the adaptation and de-adaptation phases for session I and II are given in Table 2. The AIC values from the double exponential model are consistently smaller in all cases. Thus, the double exponential model is used in the subsequent sections in order to fit the group-averaged data.

#### 3.1.2. Estimated Parameters and Confidence Intervals

The parameters and 95% confidence intervals that were obtained without the linearisation assumption and transformed to common language parameters are presented in Table 3. The parameters along with their confidence intervals obtained from the double exponential models fitted to adaptation and de-adaptation symmetry series from the two sessions are given in the Supplementary File.

In the case of adaptation, the asymmetry at the beginning of the phase in session I is larger when compared to session II with overlapping confidence intervals. This overlap suggests high degree of uncertainty in this result. The total change in symmetry is also larger in session I as compared to session II with overlap in confidence intervals. The slower exponential trends differ across the two sessions as during session I and II approximately 235 and 30 strides are taken to complete 50% of these trends, respectively. At the end of the adaptation phase, there is larger asymmetry in session II when compared to session I. Moreover, a positive overshoot is detected in session II with undefined upper confidence interval which suggests that it can not be asserted with 95% confidence that an overshoot will be observed in future data.

In the case of de-adaptation, no statistically significant difference is observed across the two sessions. Nonetheless, the initial asymmetry, total change from the beginning to the end of the phase, the number of strides taken to complete 50% of the total change in the slow and the fast trends are smaller in session II compared to session I. The asymmetry at the end of the phase is larger in session II compared to session I.

The residual standard deviation seems to be smaller for models that are fitted to session II as compared to session I for both adaptation and de-adaptation phases. To reiterate, approximately 68% of step lengths fall within a distance of one standard deviation on either side of the exponential trend fitted to the symmetry series. This result suggests that the random variability (including between-participant and error variance) across the strides decreases from session I to session II.

### 3.2. Participant Symmetry Series

**Figure 6:**
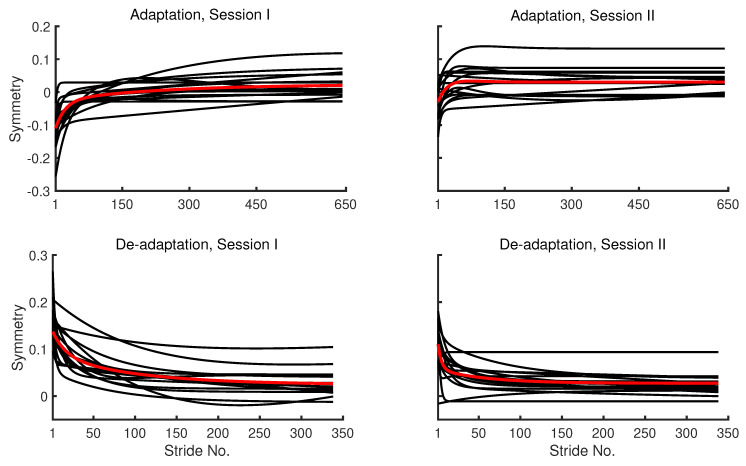
Fitted values from the double exponential models for participant (black) and group-averaged (red) symmetry series.

The fitted values from the double exponential models that are applied to participant and group-averaged symmetry series are presented in Figure 6.

The double exponential models and not the single exponential models were fitted for the sake of comparison with the models fitted to group-averaged data. However, it should be noted that the double exponential model is not favoured by the AIC criterion in all cases of the participant symmetry series, especially the ones from the second session. The residuals-vs-strides plots for the participant symmetry series are provided in the Supplementary File. These results are obtained by fully automated application of the model fitting MATLAB function to the data without any intervention. The results suggest that the algorithm is capable of successfully fitting the model to both the group-averaged and individual symmetry series. These results also suggest that the trends in symmetry series of participants generally resemble the trends in group-averaged symmetry series. A few conspicuous trends can be seen in the residuals-vs-strides plots from models fitted to participant symmetry series. First, the residuals from the double exponential models seems to have 0 mean across the strides in almost all cases. This suggests that the double exponential model explains most of the systematic trends in the data, and it is not leaving out any linear trends in the residuals. Second, the residuals seem to have cyclic trends in a large number of cases.

## 4. Discussion

To the best of our knowledge, this is the first study that has presented: (i) a transformed set of parameters with a common language interpretation for the exponential models used in split-belt treadmill training research, (ii) parameter bounds for the regression parameters to allow for nonlinear regression while using particle swarm optimisation without providing initial guess, (iii) application of existing statistical methods such as AIC and confidence intervals without the linearity assumption to split-belt treadmill training, and (iv) publicly available software for application of the aforementioned methods. The application of the proposed methods to a split-belt treadmill training dataset has also generated two novel insights. First, the participant step length symmetry series may have cyclic trends, which cannot be explained by the exponential models. Second, the group-averaged symmetry series may exhibit an overshoot. The practicalities of applying the proposed methods and the hypotheses that emerge from these insights are further discussed in the following paragraphs.

By its very nature, finding an initial guess is exploratory, tarnishes the validity of rejecting or confirming a hypothesis, and it also leaves the researcher with a sense of self-doubt as to whether they were able to make the right initial guess or not. Using the particle swarm algorithm, which employs a global approach to optimisation, the need to specify a good initial guess is eliminated. Instead, the algorithm starts from a random guess and attempts to reach the set of parameters which corresponds to the global or a near-global solution. The likelihood of convergence to the global solution is further increased by running the algorithm twice starting at different random guesses. However, we must acknowledge that there are still some limitations of the proposed optimisation approach. First, with a future dataset, the weight parameters in the cost function for the double exponential model (refer to Equation (6)) may be sensitive to population characteristics. We propose that this problem can be detected by randomly choosing one or two symmetry series from the larger dataset, running the model fitting function on these series multiple times, and checking whether the function returns different results each time. By appropriately changing the weight parameters, the sensitivity of the algorithm to initial conditions may be diminished. Second, before fitting an exponential model, the overall trend is detected while using the mean difference of first and last 50 strides (refer to Section 2.3.1). This rule based on 50 strides may not work in every case. In fact, it failed to detect the correct trend in two out of 30 participant symmetry series. Therefore, caution is warranted while using this rule in future studies. A more robust, yet computationally expensive method is to fit the models with bounds for both the positive and negative trend, and choose the solution with the smaller cost.

Visual analysis of residuals not only allowed the evaluation of the model assumptions, but it also revealed the nature of the portion of data that the model failed to explain. For example, some previous studies have concluded that the double exponential model is better than the single exponential model, as it achieves a higher $R^{2}$ value. In this report, the residuals analysis not only confirmed these findings but also highlighted the fact that the single exponential model fails, because it leaves out the initial fast dynamics in the data (refer to Figure 5) which seem to have a distinct exponential trend as compared to the overall long-term trend. In the past, some researchers have also attempted to fit exponential models to participant symmetry series and have reported very low $R^{2}$ values when compared to those for group-averaged data [41]. Partly, this is due to averaging across participants which reduces the variance in the data. However, the residuals analysis for the participant symmetry series in this report suggests that the findings of these past studies may also be explained by the fact that at the participant level cyclic trends in the data are too dominant to be ignored. As the exponential models do not explain oscillating behaviour, these cyclic trends are passed to the residuals. These trends suggest that the performance of the participants oscillate over time and they need to be modelled with sinusoids along with the exponential trends or by using an autoregressive variance structure instead of constant variance across strides for residuals [78].

Whilst the analysis of residuals in itself is a valid and adequate method for assessing and comparing the explanatory capability of the fitted models, a quantitative measure for the goodness of the fit adds to the exactitude of conclusions. To this end, we have proposed the use of AIC, which approximates the relative information lost as a consequence of using a particular model. As it is a relative measure, it can only be used to compare models fitted to the same data. In the case of group-averaged data, like the residual analysis, the AIC values also favoured the double exponential models in all cases. In the case of participant symmetry series, the double exponential model was not favoured in all of the cases by AIC. This suggests two hypotheses. First, the trends in some of the participant series are simpler and can be explained adequately by a single exponential model. Second, the cyclic trends seen in the residuals are heavily dominant, such that both the single and double exponential models are equally helpless in explaining these trends and the fitted double exponential models have higher AIC values, as they are penalised for using a larger number of parameters to explain the same amount of variance in the data. These hypotheses need to be tested in future research in combination with improvements to the model, such as the addition of an autoregressive variance structure to cater to the cyclic trends.

The inference in the past studies was based on a comparison of confidence intervals from models fitted to group-averaged data. We have proposed the use of a method that results in more exact confidence intervals. A comparison (please refer to the Supplementary File) of its results with the results of the traditional method suggests that the latter fails in some cases due to highly nonlinear behaviour of the fitted model, which cannot be approximated with linearisation. Thus, the proposed method will improve the inferences that are drawn from the nonlinear models fitted to symmetry series in future studies. In some of the past studies [41], inferences were drawn for group differences by comparing the means of the regression parameters from the exponential models fitted to the individual participants in those groups. The means were compared using a linear model (*t*-test). The methods proposed in this report can also be used for this type of two stage analysis. However, based on the results of residual analysis for the models fitted to participant symmetry series, we suggest that the future researchers should exercise caution to make sure that the models fitted to the participant data have adequate fit before using the two stage inference method. Yet another approach, which combines this two stage process into a single statistical model, is the nonlinear mixed effects regression [78,79]. Although further work is required to nut out the finer details of this approach, we propose that a mixed nonlinear regression model may be setup, which fits exponential trends to participant symmetry series and jointly regresses group means under the assumption that the participants in these groups are randomly drawn from the populations that these groups represent. With such a model, it may be easier to observe and correct for any systematic trends in the residuals.

In this study, the inferences are drawn on the common language parameters that were obtained from the estimated parameters of the exponential models fitted to the data. The finding that the participants took a smaller number of strides to complete 50% of the adaptation in session II compared to session I is inline with the past research [80]. However, the finding that the participants at the end of the adaptation phase in session II had higher asymmetry when compared to session I is in contrast with the previous research in which a similar trend was seen but the difference was not statistically significant [80]. Based on the fact that one adapts/de-adapts to their baseline values, higher asymmetry could be because of a lack of full de-adaptation as the de-adaptation phase lasted for only 10 min. as opposed to 15 min. of adaptation phase during session I. Therefore, during session II, the individuals may have adapted to their new baseline values. The higher asymmetry may also be explained by the hypothesis that in this study, the participants held onto a safety handrail throughout the adaptation phase where, as in the past study, they did not do so, and holding onto a safety handrail has been found to affect the response of participants in split-belt treadmill training [80,81]. The application of the proposed exponential models also revealed that there was an overshoot in the symmetry series from the adaptation phase in session II. To the best of our knowledge, this is the first study in which the phenomenon of overshoot is observed while using the exponential models. In terms of motor training, this overshoot may be interpreted as a result of the overcompensation by the error correcting neurophysiological mechanisms that are responsible for directing gait. Further research is required to elucidate the mechanisms that may explain this overshoot.

Non-parametric plateau-based methods for evaluating rate of adaptation and de-adaptation have also been proposed in the past research [82,83]. These methods evaluate strides to steady-state symmetry as the number of strides taken to reach symmetry values that remain within a specified number of standard deviations of the mean of last 30–100 strides. The application of these methods is more straight forward compared to the exponential models as they are not based on nonlinear regression. However, their limitation is the arbitrary selection of parameters, such as the number of standard deviations and the inability to fully model the data to result in intuitive parameters such as initial asymmetry, final symmetry, etc. Moreover, these methods couple the rate of adaptation and de-adaptation with the variability in the symmetry series in the last 30–100 strides. For example, the plateau methods will report a larger number of strides to steady-state for a symmetry series that has a smaller variability in the last 30–100 strides. We hope that the exponential models proposed in this report not only overcome these limitations, but are also easier to apply.

Finally, the methods that are proposed in this report are strictly in accordance with the specific needs for fitting exponential trends in step length symmetry series. However, the researchers interested in studying other outcome measures for motor training by fitting nonlinear exponential models can easily adapt the proposed procedures [84,85,86,87]. We propose that, in most of the cases, a modification to the parameter bounds may be the only change required. This change can also be avoided by applying a suitable normalisation to the outcome measure, so that it stays within [−1, 1].

## 5. Conclusions

Whilst the nonlinear regression literature has developed substantially over time, the current methods used for nonlinear regression of single and double exponential models to trends in split-belt treadmill training tasks are limited by the specification of initial guess for regression parameters, inadequacy of the coefficient of determination for nonlinear model, and inference that is based on the untested assumption that the fitted model has a good linear approximation. In this research, we have attempted to adapt statistical methods for split-belt treadmill training which address these limitations. Publicly available software that is based on the proposed methods is also provided, which fully automates nonlinear regression for exponential trends in split-belt treadmill training.

## Figures and Tables

**Table 1:** Summary of parameter bounds for the single and double exponential models. Direction is defined as positive (+ve) or negative (−ve) depending on the direction from which the symmetry series approaches its final value.

Model (Parameters)	Direction	Overshoot	Bounds
Single (*a*; *b*; *c*)	+ve	–	[0, 2]; [−ln(2), 0]; [−1, 1]
	−ve	–	[−2, 0]; [−ln(2), 0]; [−1, 1]
Double ($a_{s}$; $b_{s}$; $a_{f}$; $b_{f}$; *c*)	+ve	No	[0, 1]; [−ln(2), 0]; [0, 1]; [−ln(2), 0]; [−1, 1]
		Yes	[−1, 0]; [−ln(2), 0]; [0, 1]; [−ln(2), 0]; [−1, 1]
	−ve	No	[−1, 0]; [−ln(2), 0]; [−1, 0]; [−ln(2), 0]; [−1, 1]
		Yes	[0, 1]; [−ln(2), 0]; [−1, 0]; [−ln(2), 0]; [−1, 1]

**Table 2:** Akaike’s information criterion (AIC) values for the single and double exponential models.

Phase	Session	AIC (Single Exp. Model)	AIC (Double Exp. Model)
Adaptation	I	−2017.63	−2327.59
	II	−2472.15	−2496.23
De-adaptation	I	−1326.61	−1371.21
	II	−1409.85	−1461.28

**Table 3:** Common language parameters along with their 95% confidence intervals (CIs) rounded to three decimal places from the double exponential models. Note: UD stands for undefined, std. stands for standard deviation and * denotes statistical significance.

Phase	Parameter	Session		CI Overlap
		Session I	Session II	
Adapt	Asymmetry at beginning	−0.114 [−0.126, −0.106]	−0.033 [−1.018, 0.888]	Yes
	Total change	−0.137 [−0.148, −0.133]	−0.063 [−1.048, 0.857]	Yes
	Strides to 50% changes	235 [194, 299]; 21 [18, 24]	30 [25, 52]; 28 [19, 32]	No *, Yes
	Asymmetry at end	0.024 [0.021, 0.027]	0.030 [0.030, 0.031]	No *
	Overshoot	UD [UD, UD]	0.034 [0.030, UD]	Yes
	Residuals std.	6.386 × 10^−3^	5.600 × 10^−3^	–
De-adapt	Asymmetry at beginning	0.141 [−0.863, 0.227]	0.125 [0.090, 0.154]	Yes
	Total change	0.117 [0.080, 0.200]	0.098 [0.070, 0.125]	Yes
	Strides to 50% changes	100 [76, 13203]; 15 [8, 34]	58 [38, 252]; 4 [2, 11]	Yes, Yes
	Asymmetry at end	0.024 [−0.943, 0.027]	0.027 [0.019, 0.029]	Yes
	Overshoot	UD [UD, UD]	UD [UD, UD]	Yes
	Residuals std.	7.043 × 10^−3^	6.161 × 10^−3^	–

## Supplementary Materials

The following are available online at https://www.mdpi.com/2076-3425/10/10/737/s1, Fit and diagnostic information for group-averaged and participant symmetry series.

## Abbreviations

The following abbreviations are used in this manuscript:

AIC	Akaike’s information criterion
PSO	Particle swarm optimisation
SS	Same speed configuration
DS	Differential speed configuration
ctDCS	Cerebellar transcranial direct current stimulation
AUTEC	Auckland University of Technology Ethics Committee
CIs	Confidence intervals
UD	Undefined
