# Supplementary material for: On Nonlinear Regression for Trends in Split-Belt Treadmill Training
Source: Brain Sci. 2020 Oct 14;10(10):737. doi: 10.3390/brainsci10100737 (PMC7602156; doi:10.3390/brainsci10100737)

Supplementary File: “On Nonlinear Regression for Trends in Split-belt Treadmill Training”

Usman Rashid
09/07/2020

Section 3.1.1: Fit and diagnostic plots for group-averaged data

Session II, Adaptation

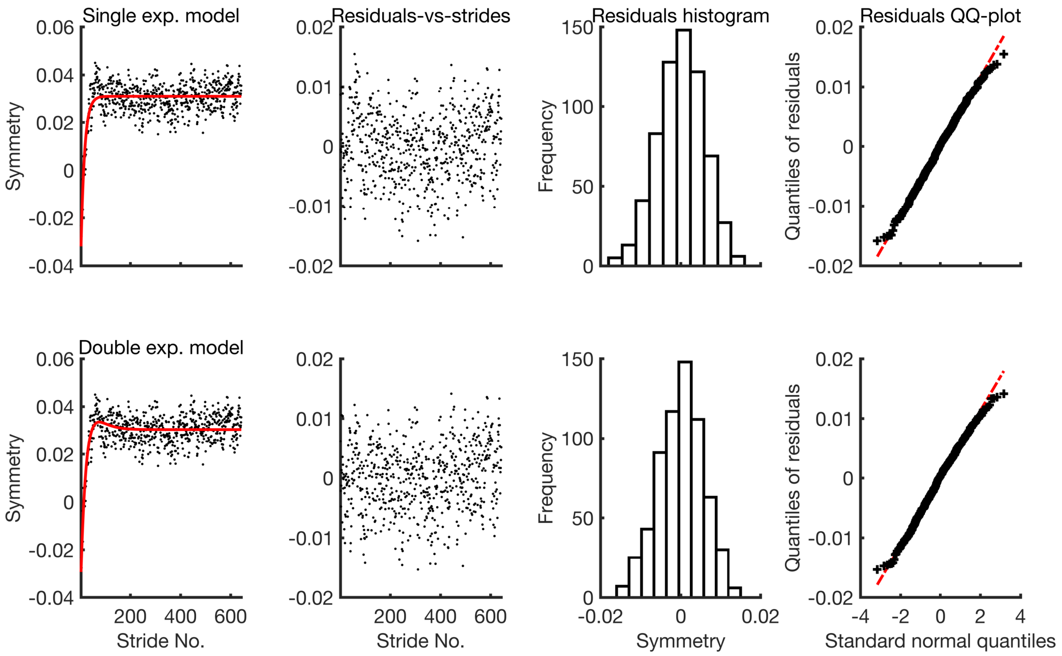

Session I, De-adaptation

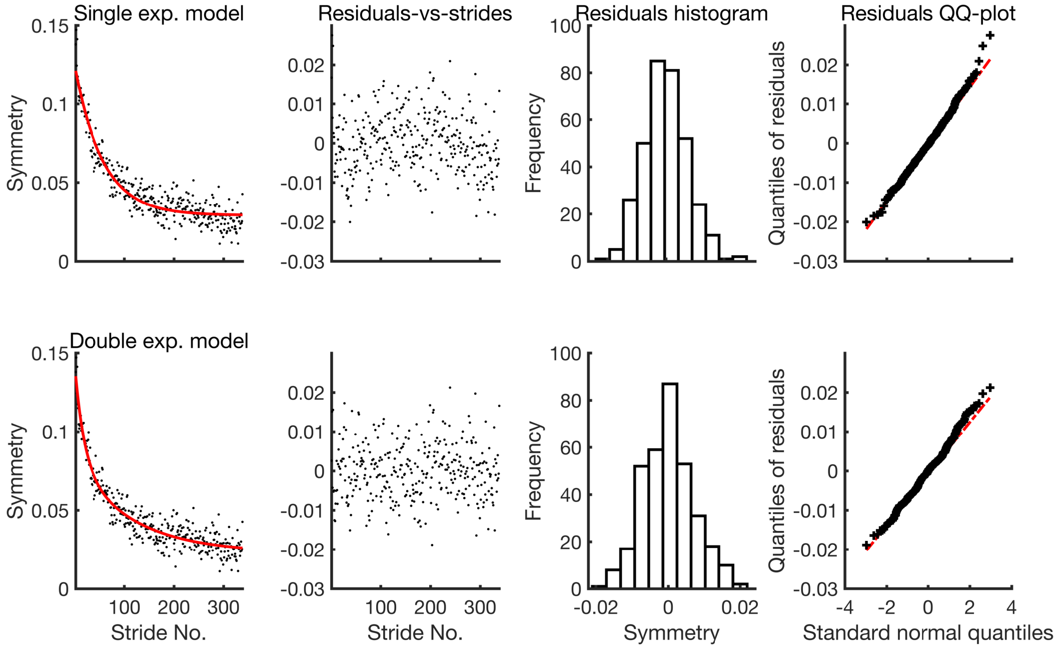

Session II, De-adaptation

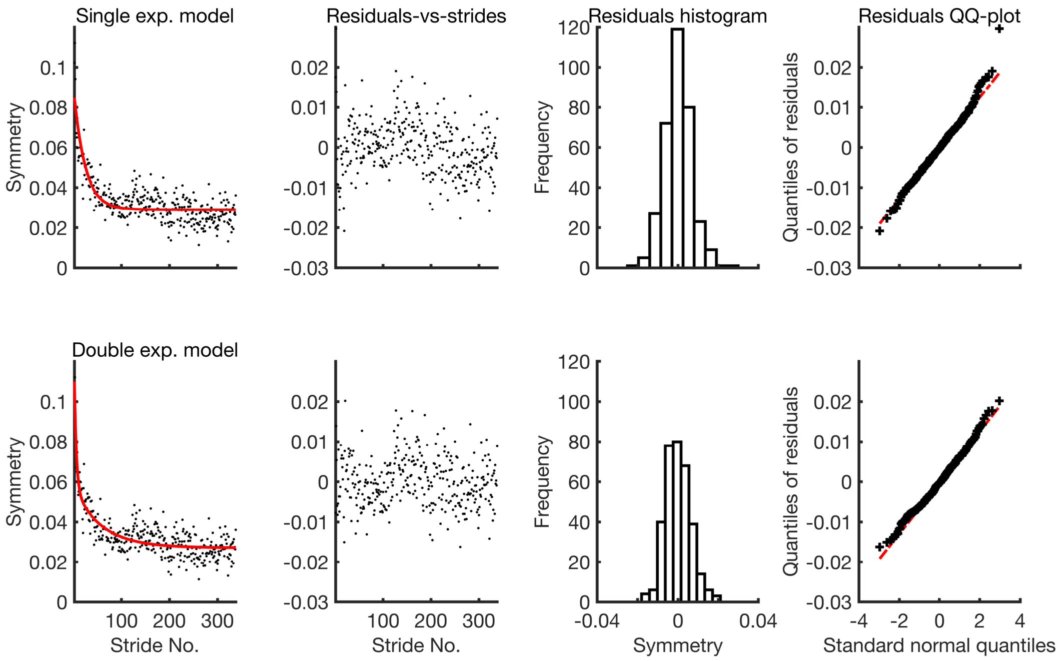

Section 3.1.2: Estimates and 95% confidence intervals without the linearisation assumption. All the numbers are rounded to three decimal places.

Phase	Parameter	Session		CI Overlap
		Session I	Session II	
Adapt	$\hat{a}_s$	-0.050 [-0.054, -0.046]	0.641 [0.021, 0.942]	No*
	$\hat{b}_s$	-0.004 [-0.005, -0.003]	-0.033 [-0.039, -0.019]	No*
	$\hat{a}_f$	-0.088 [-0.094, -0.087]	-0.704 [-1.069, -0.084]	Yes
	$\hat{b}_f$	-0.047 [-0.054, -0.041]	-0.035 [-0.051, -0.031]	Yes
	$\hat{c}$	0.024 [0.021, 0.027]	0.030 [0.030, 0.031]	No*
De-adapt	$\hat{a}_s$	0.063 [0.041, 0.134]	0.030 [0.018, 0.039]	No*
	$\hat{b}_s$	-0.010 [-0.013, 0.000]	-0.017 [-0.026, -0.004]	Yes
	$\hat{a}_f$	0.053 [0.039, 0.066]	0.068 [0.052, 0.086]	Yes
	$\hat{b}_f$	-0.064 [-0.116, -0.029]	-0.239 [-0.401, -0.087]	Yes
	$\hat{c}$	0.024 [-0.943, 0.027]	0.027 [0.019, 0.029]	Yes

Section 3.1.2: Estimates and 95% confidence intervals with the linearisation assumption. All the numbers are rounded to three decimal places.

Phase	Parameter	Session		CI Overlap
		Session I	Session II	
Adapt	$\hat{a}_s$	-0.05 [-0.054, -0.046]	0.641 [-224.692, 225.973]	Yes
	$\hat{b}_s$	-0.004 [-0.005, -0.003]	-0.033 [-0.384, 0.317]	Yes
	$\hat{a}_f$	-0.088 [-0.094-0.081]	-0.704 [-226.033, 224.625]	Yes
	$\hat{b}_f$	-0.047 [-0.054, -0.039]	-0.035 [-0.384, 0.313]	Yes
	$\hat{c}$	0.024 [0.021, 0.027]	0.03 [0.030, 0.031]	No*
De-adapt	$\hat{a}_s$	0.063 [0.050, 0.076]	0.030 [0.024, 0.036]	No*
	$\hat{b}_s$	-0.010 [-0.013, -0.007]	-0.017 [-0.022, -0.012]	Yes
	$\hat{a}_f$	0.053 [0.039, 0.068]	0.068 [0.052, 0.083]	Yes
	$\hat{b}_f$	-0.064 [-0.094, -0.035]	-0.239 [-0.333, -0.145]	No*
	$\hat{c}$	0.024 [0.021, 0.028]	0.027 [0.026, 0.028]	Yes

Section 3.2: Difference in AIC values from the double and single exponential models for participant symmetry series.

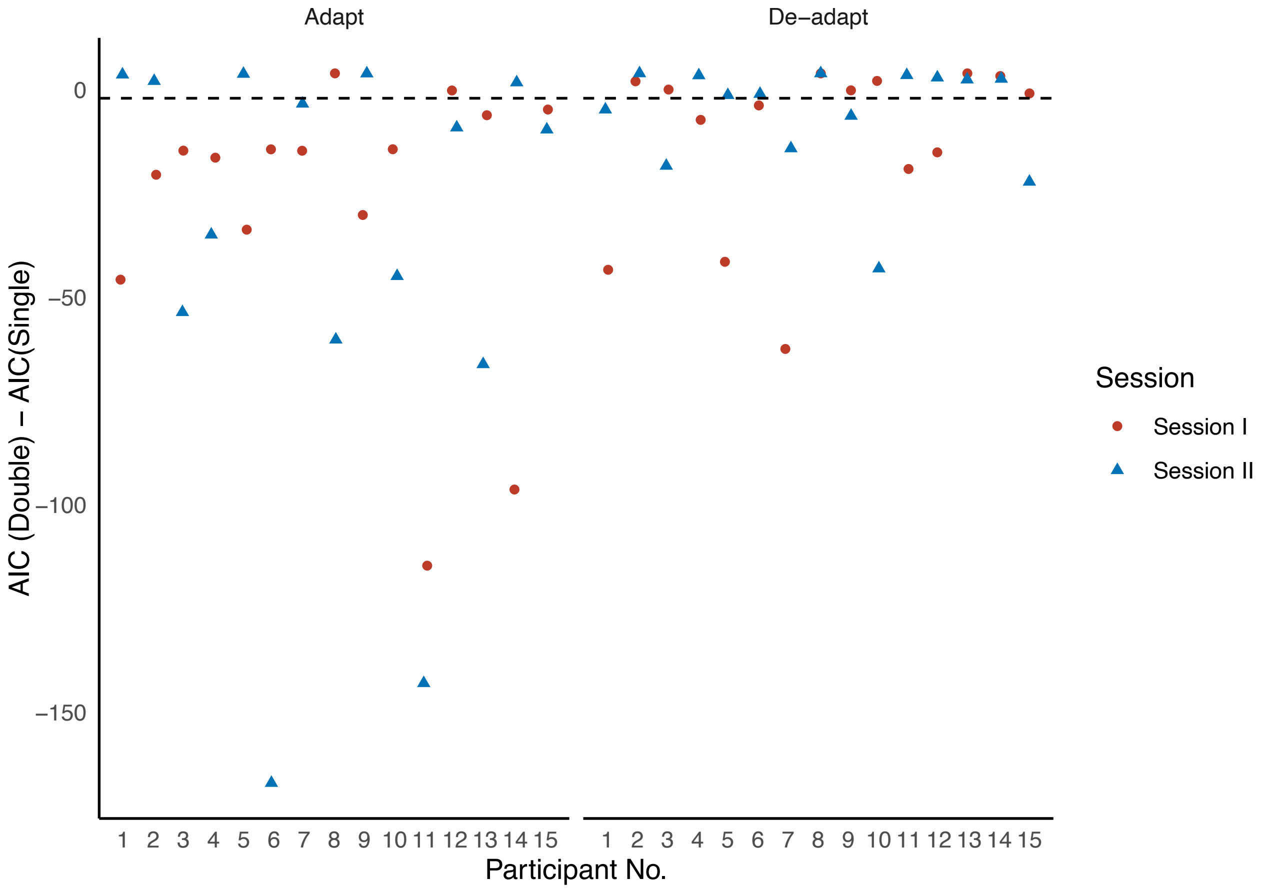

Note: The horizontal dotted line is plotted at -2.

Section 3.2: Double exponential fit and residuals-vs-strides plots for individual symmetry series

Session I, Adaptation

Participant 01, 02

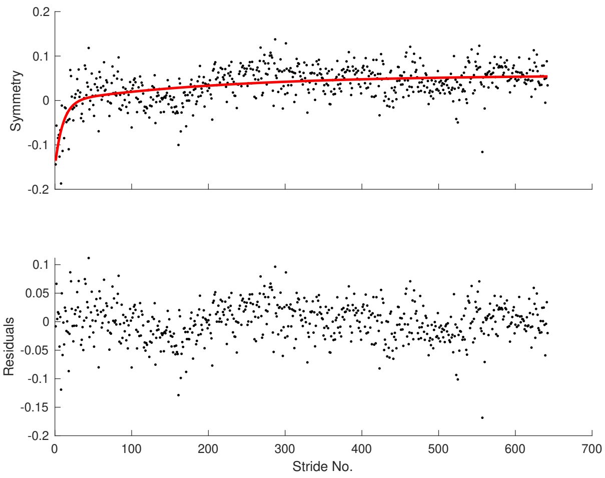

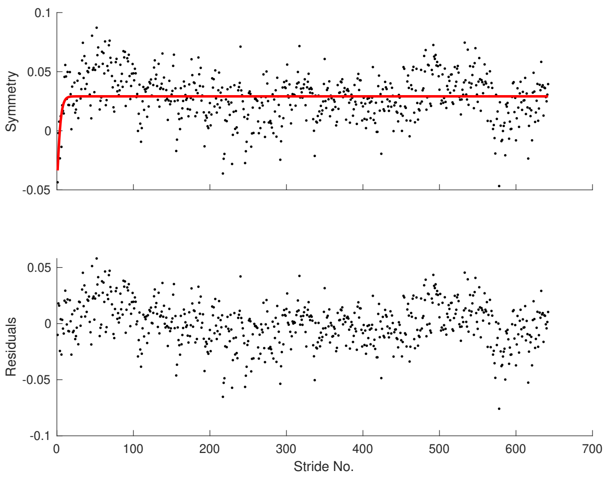

Participant 03, 04

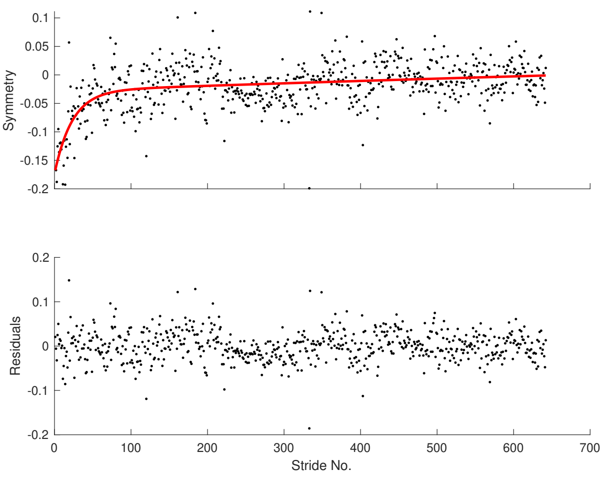

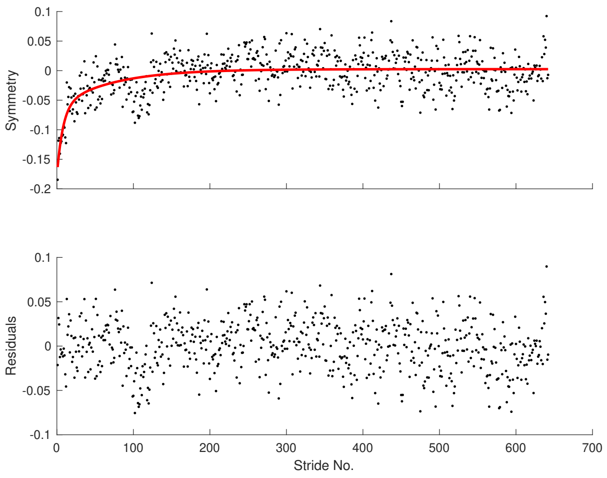

Participant 05, 06

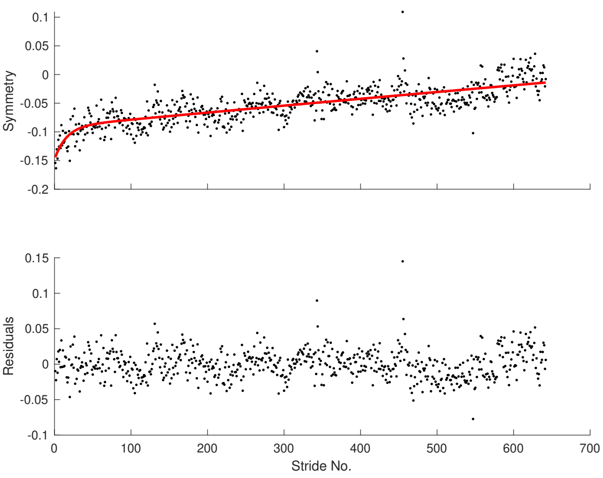

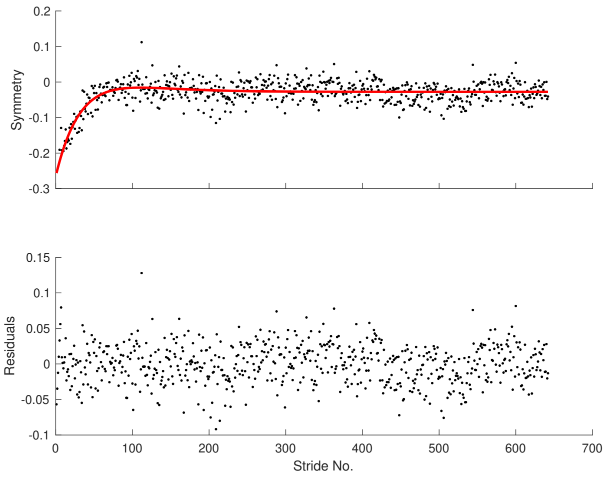

Participant 07, 08

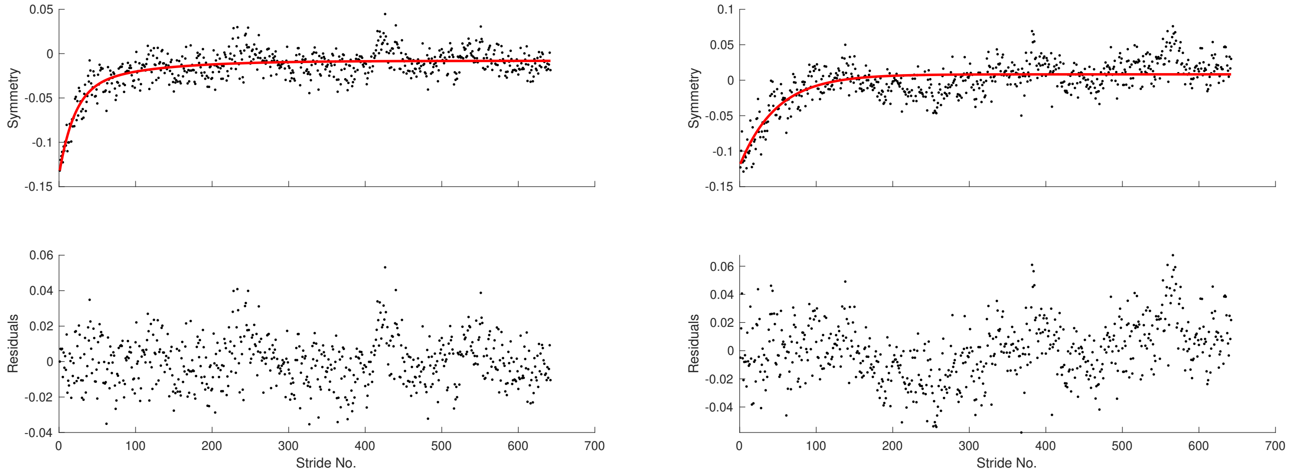

Participant 09, 10

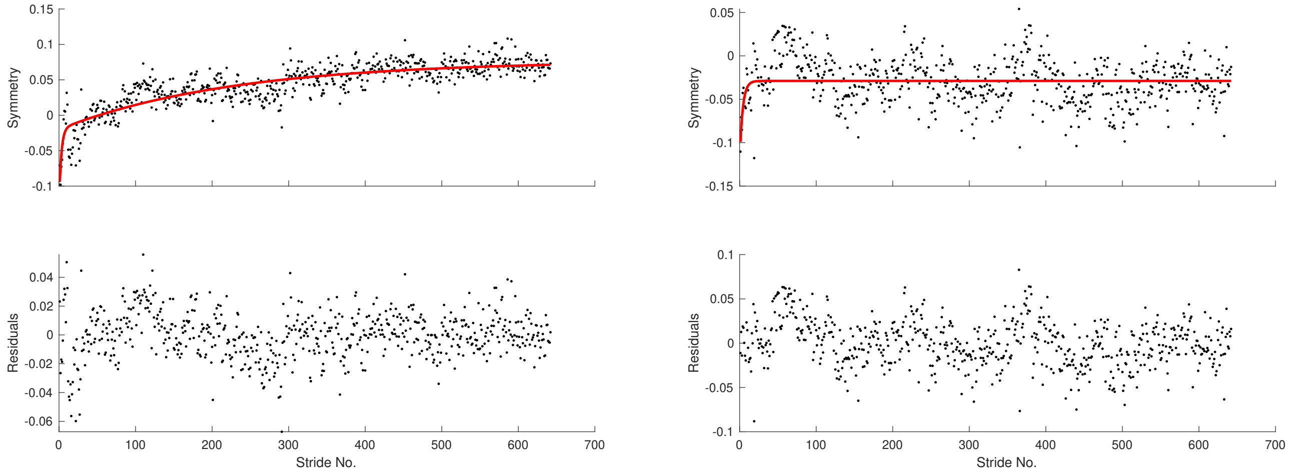

Participant 11, 12

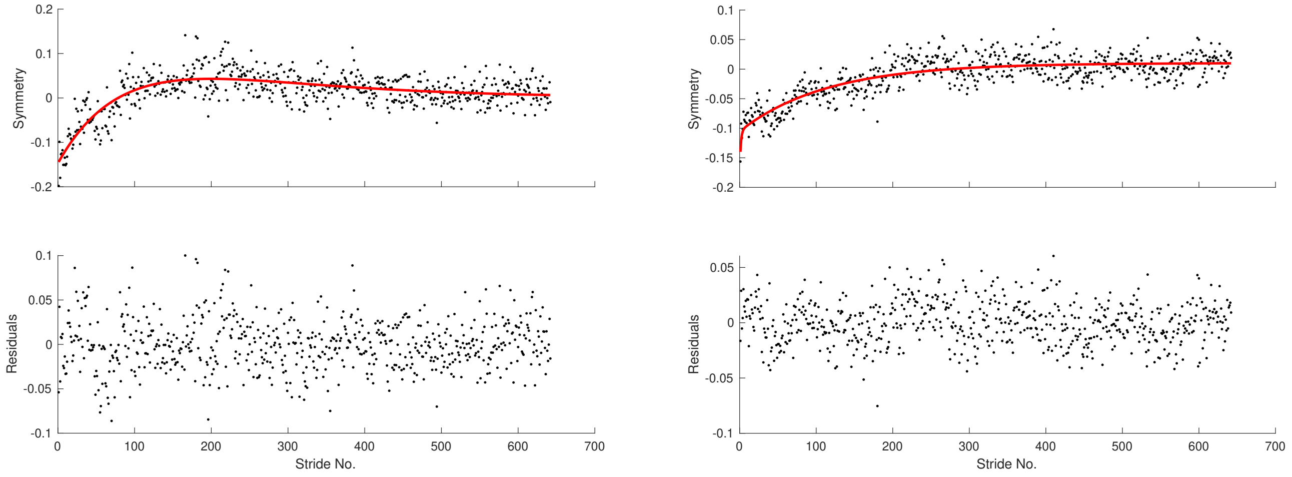

Participant 13, 14

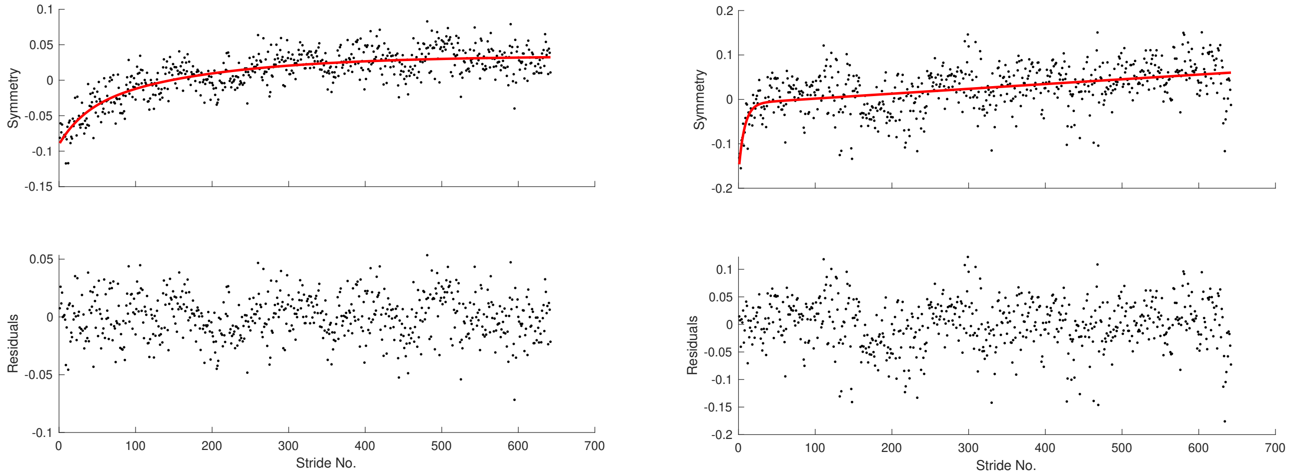

Participant 15

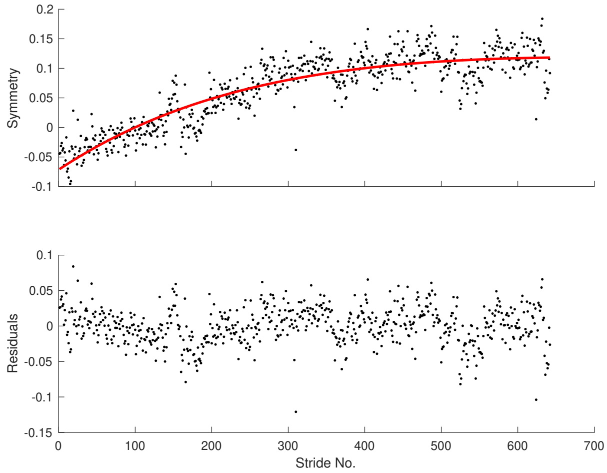

Session II, Adaptation

Participant 01, 02

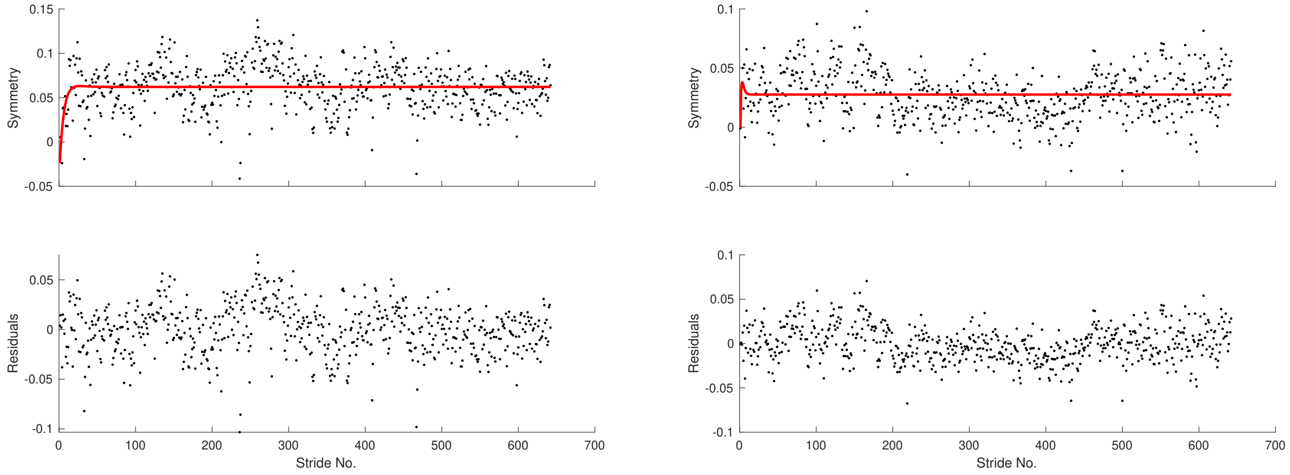

Participant 03, 04

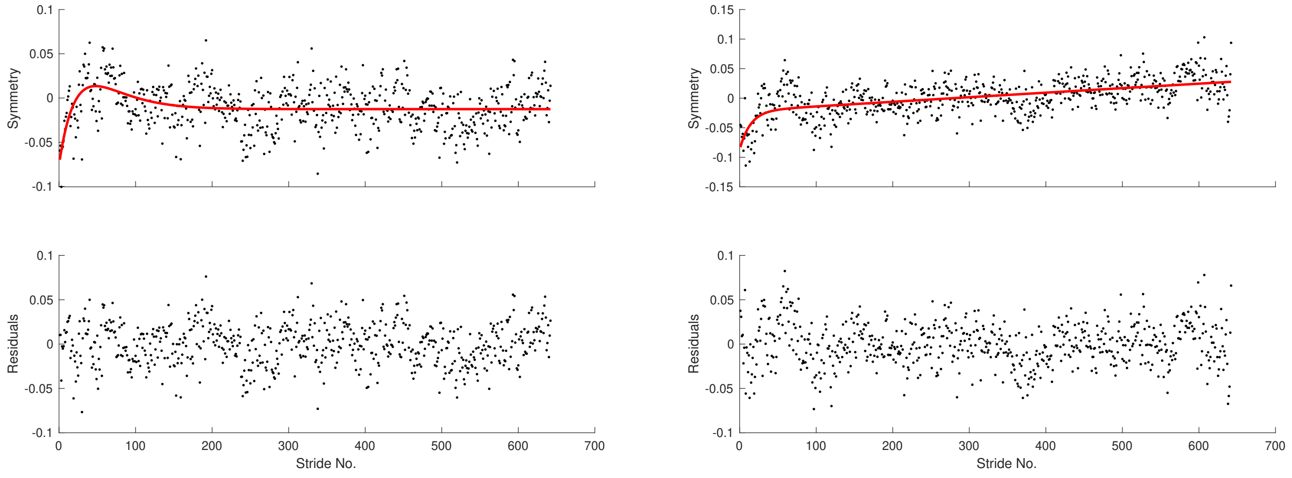

Participant 05, 06

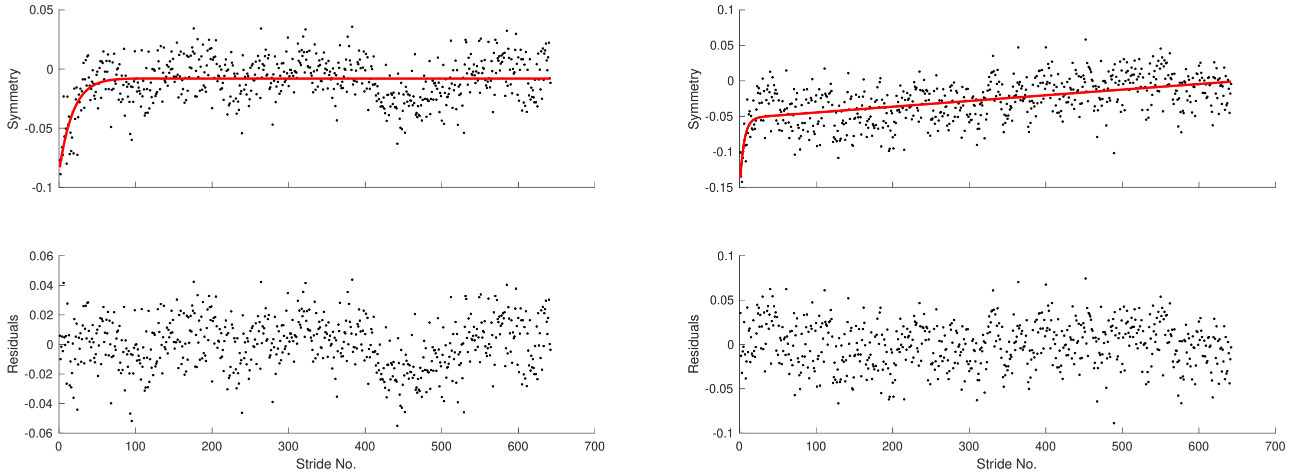

Participant 07, 08

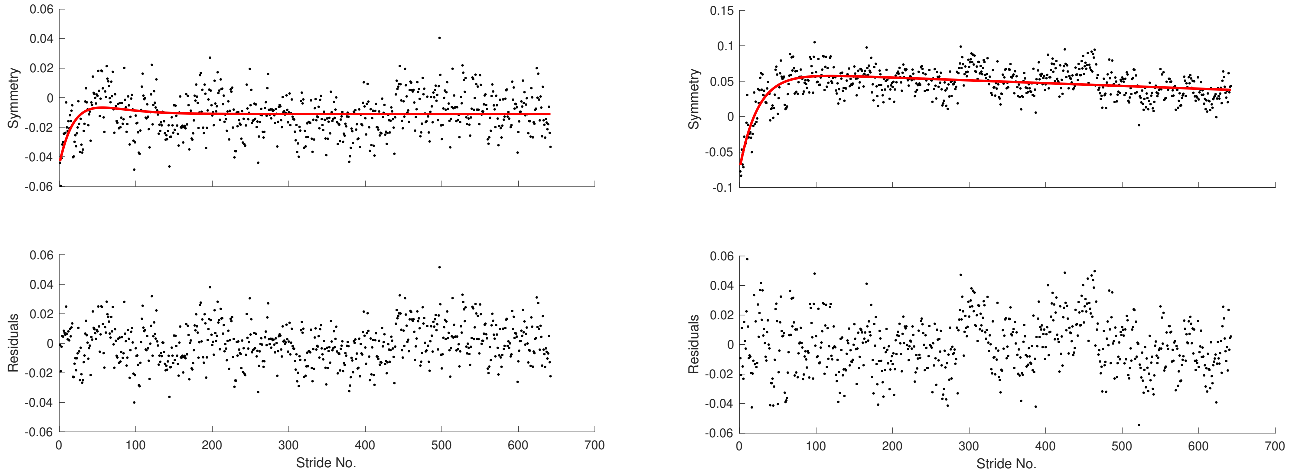

Participant 09, 10

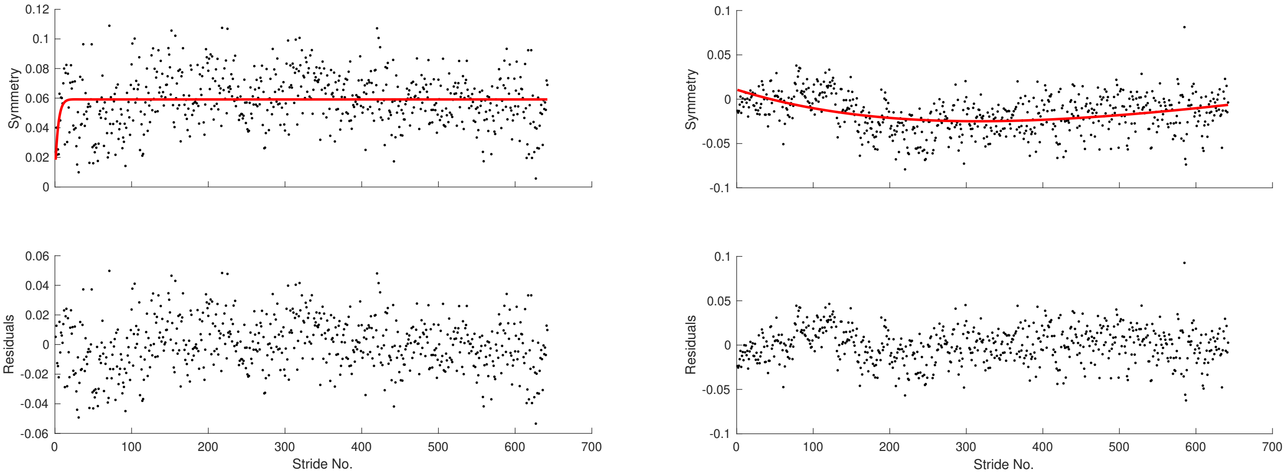

Participant 11, 12

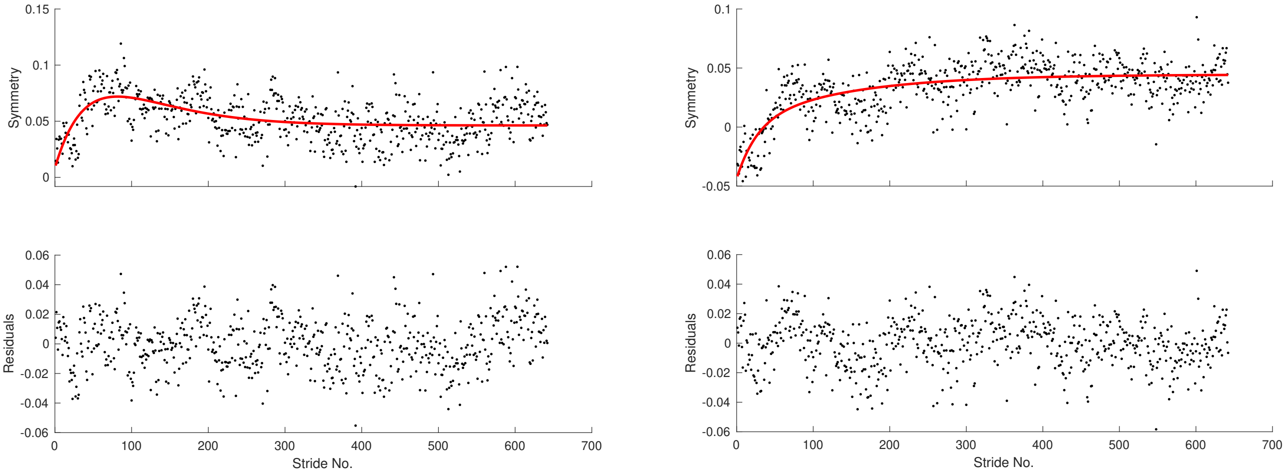

Participant 13, 14

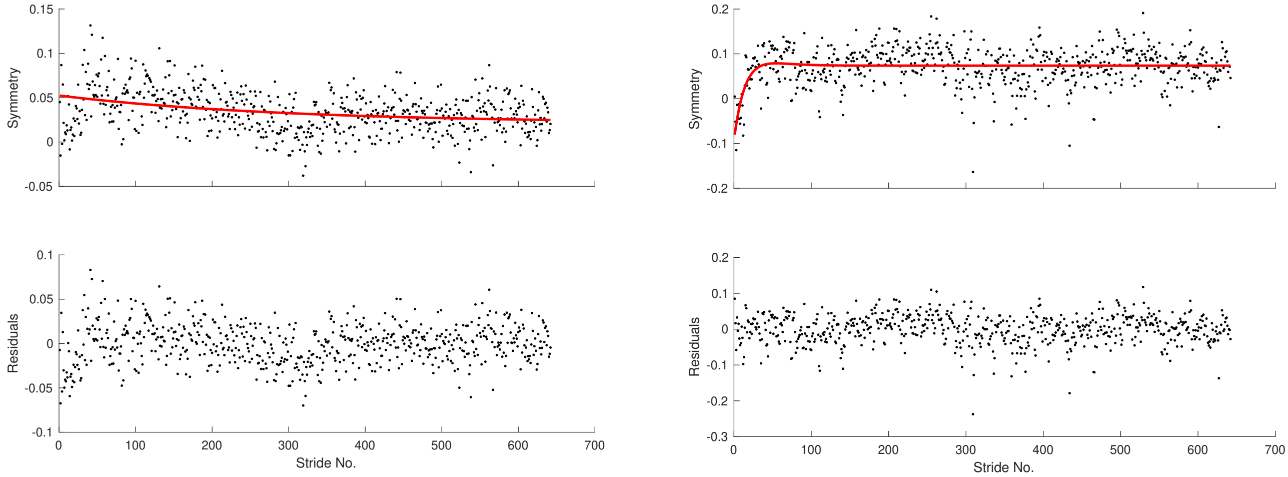

Participant 15

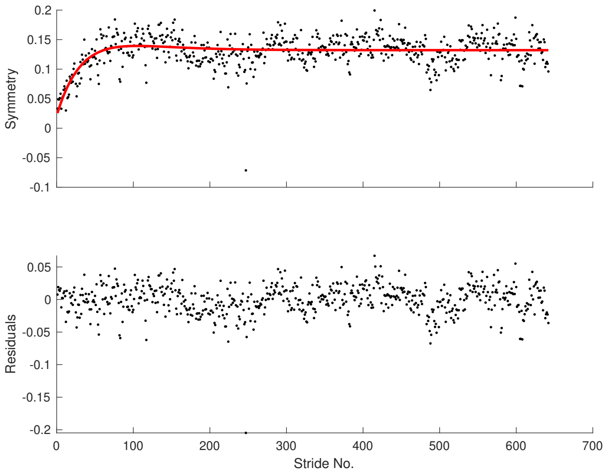

Session I, De-adaptation

Participant 01, 02

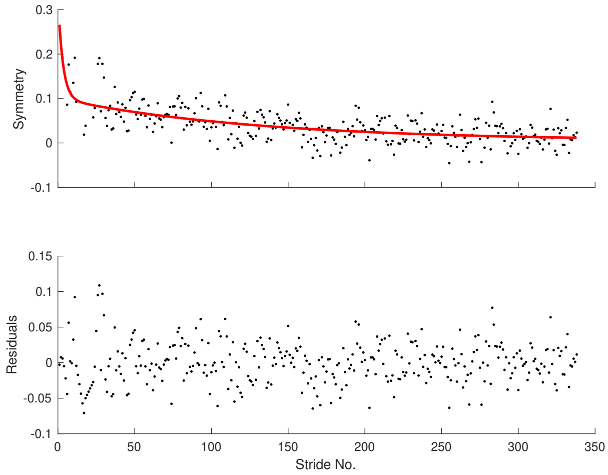

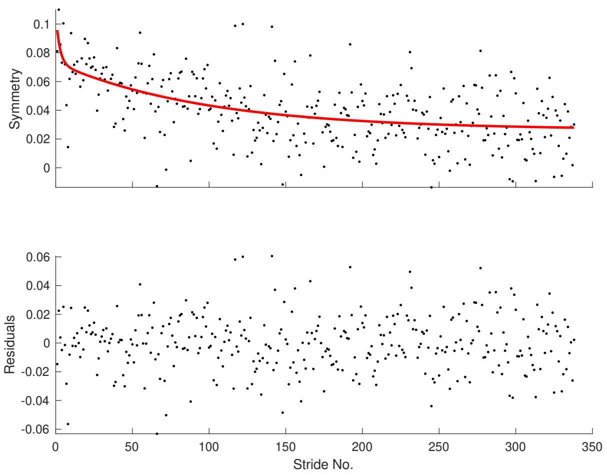

Participant 03, 04

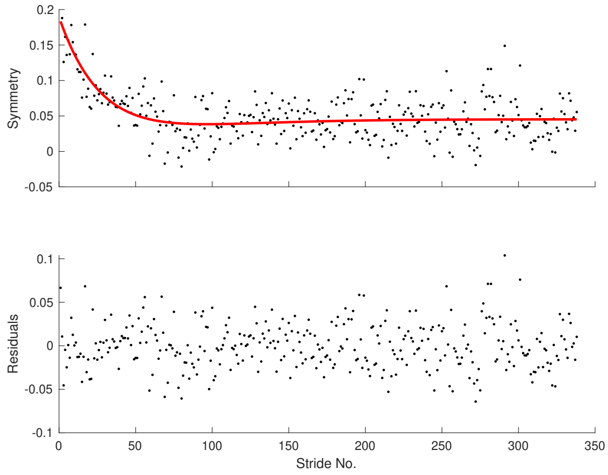

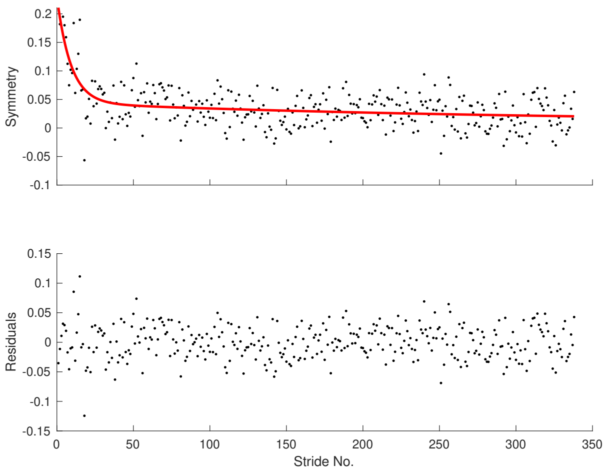

Participant 05, 06

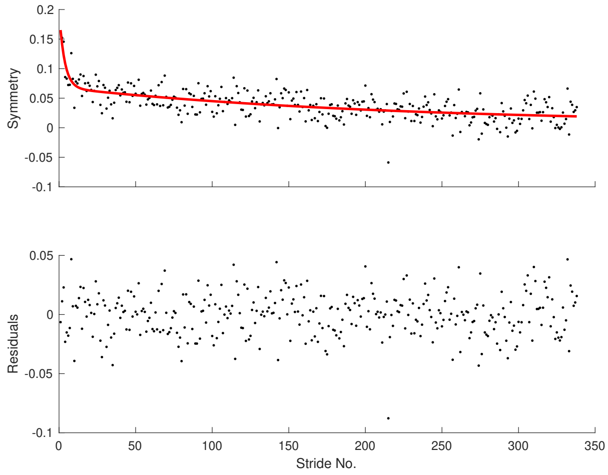

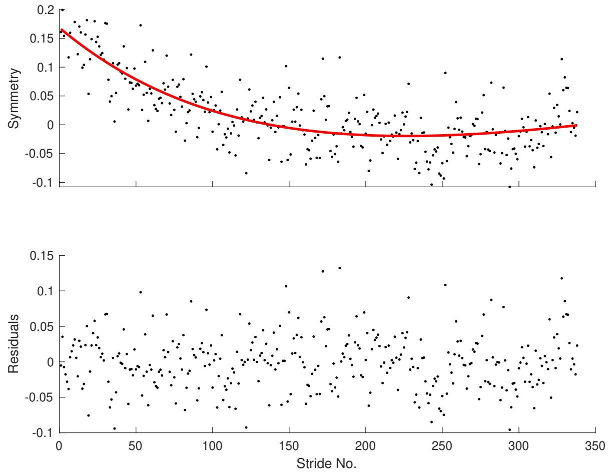

Participant 07, 08

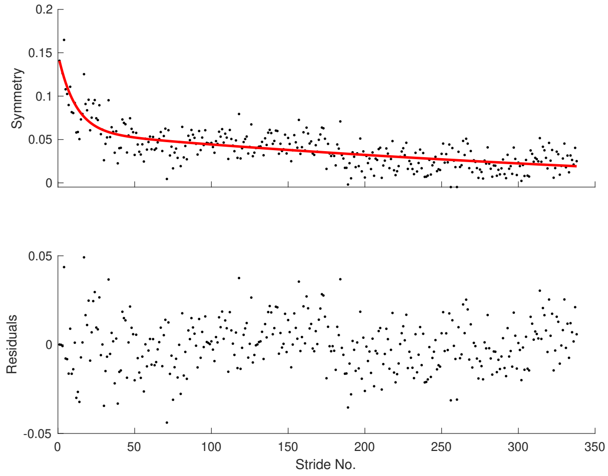

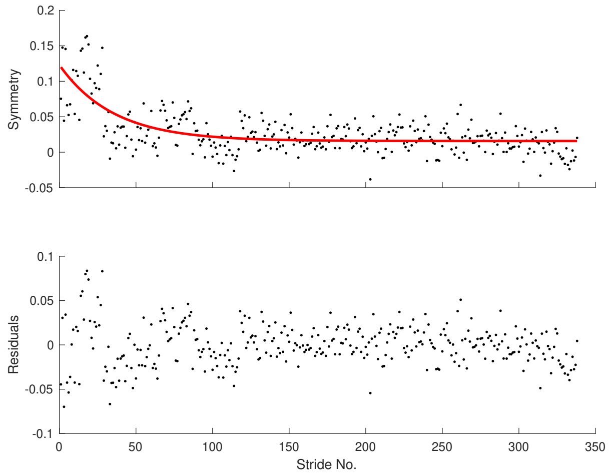

Participant 09, 10

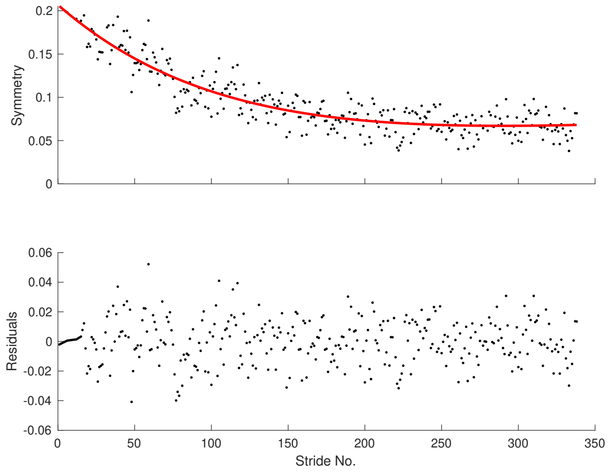

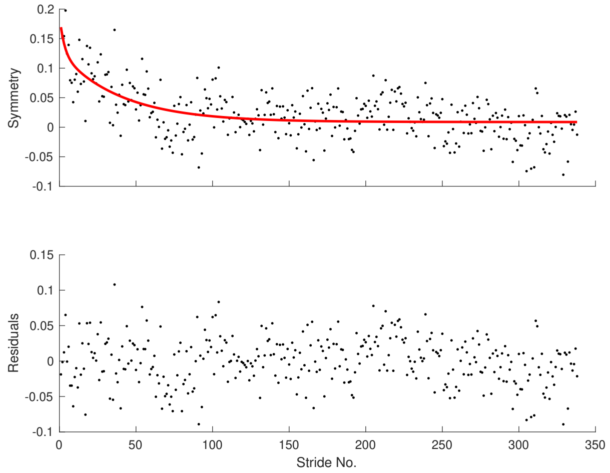

Participant 11, 12

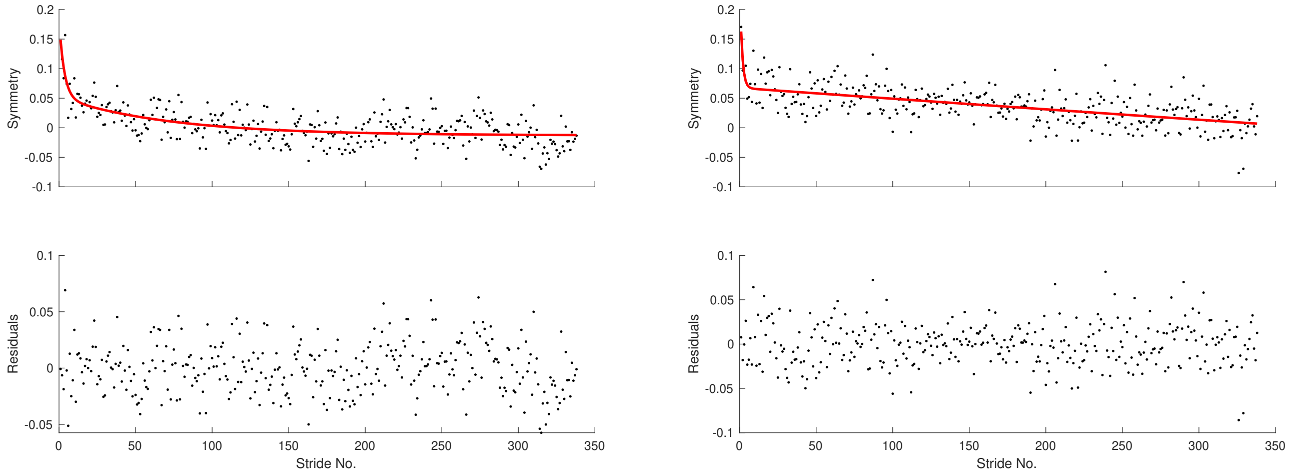

Participant 13, 14

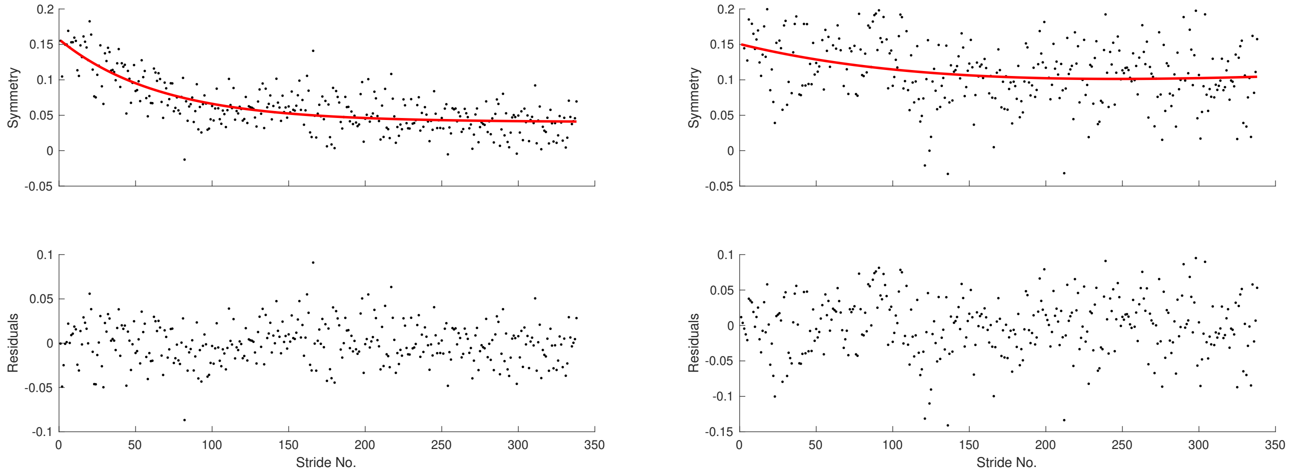

Participant 15

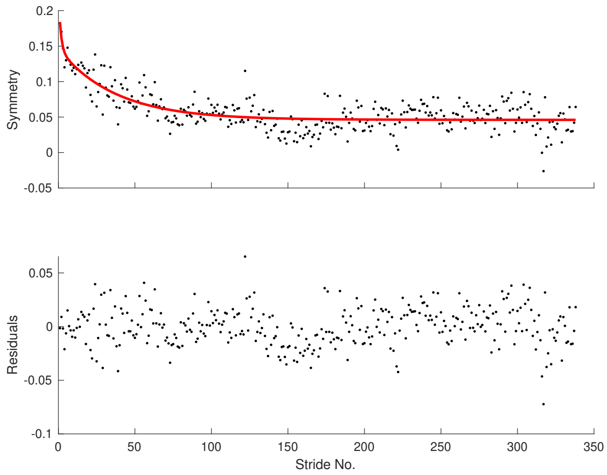

Session II, De-adaptation

Participant 01, 02

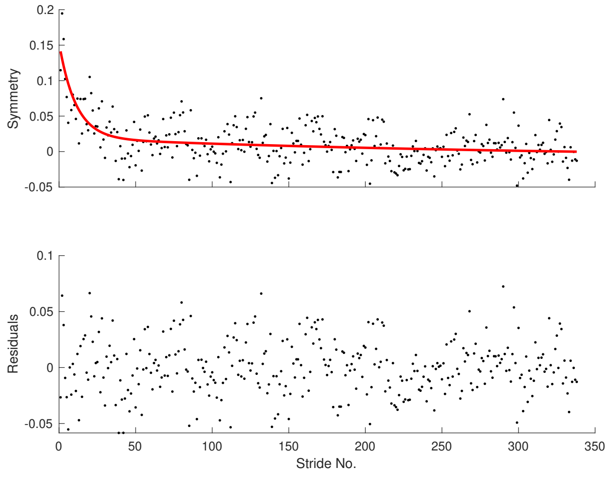

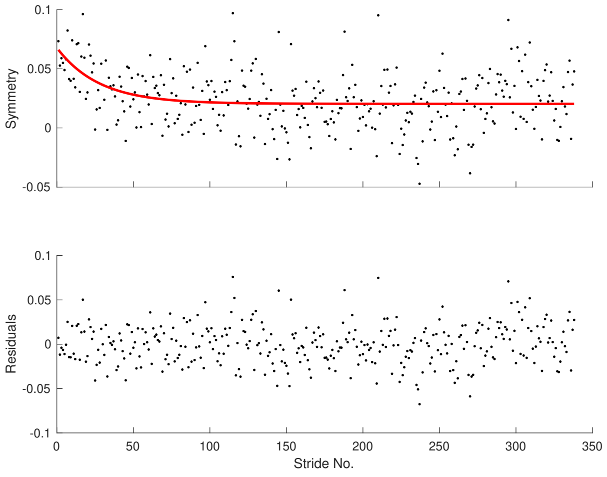

Participant 03, 04

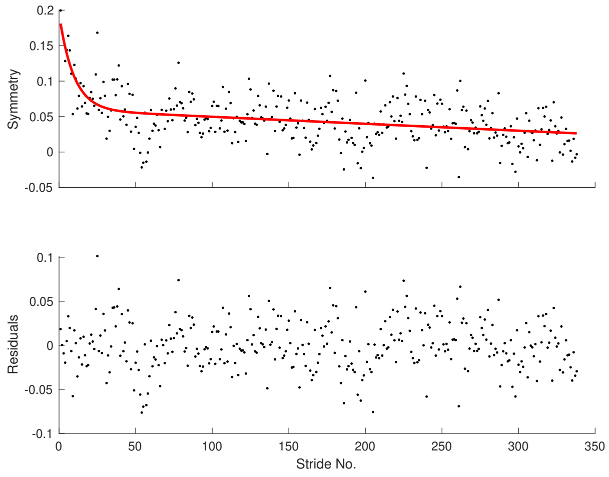

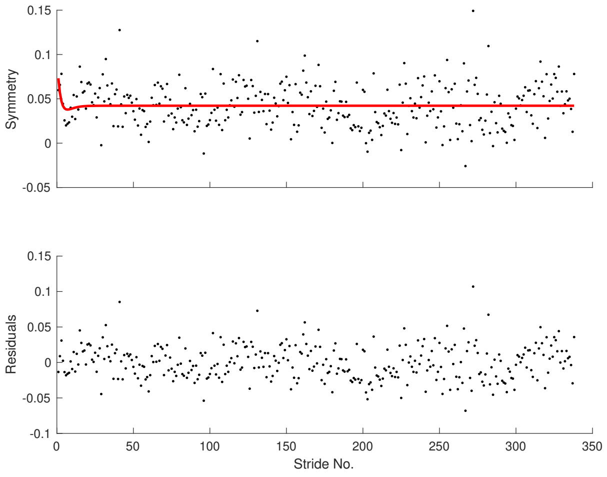

Participant 05, 06

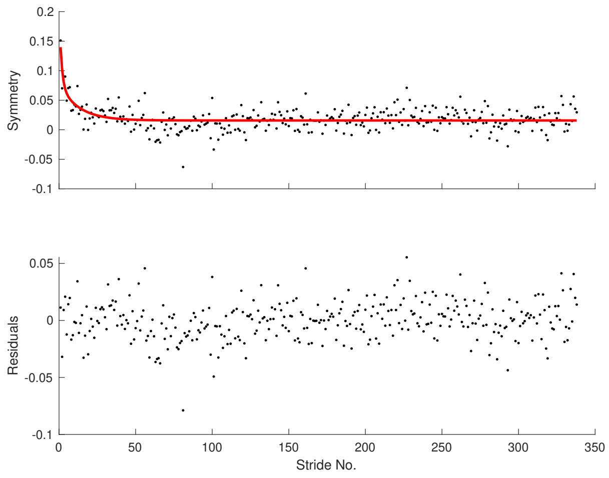

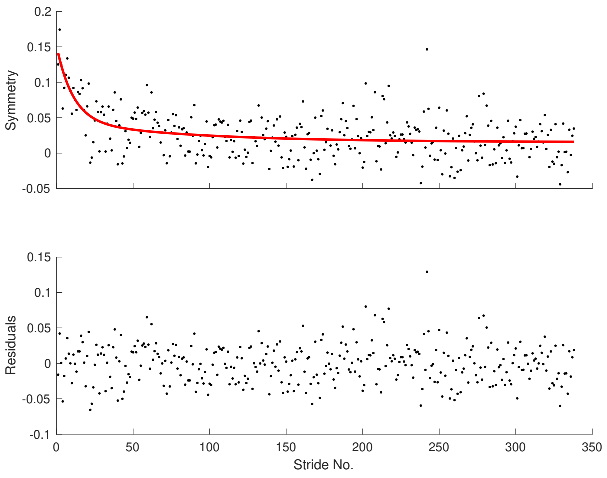

Participant 07, 08

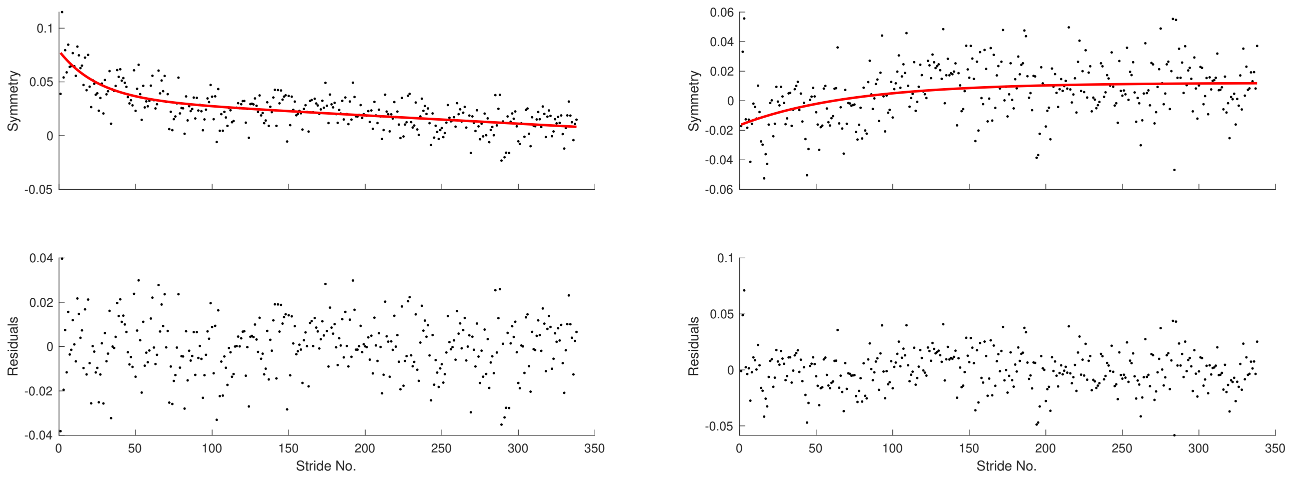

Participant 09, 10

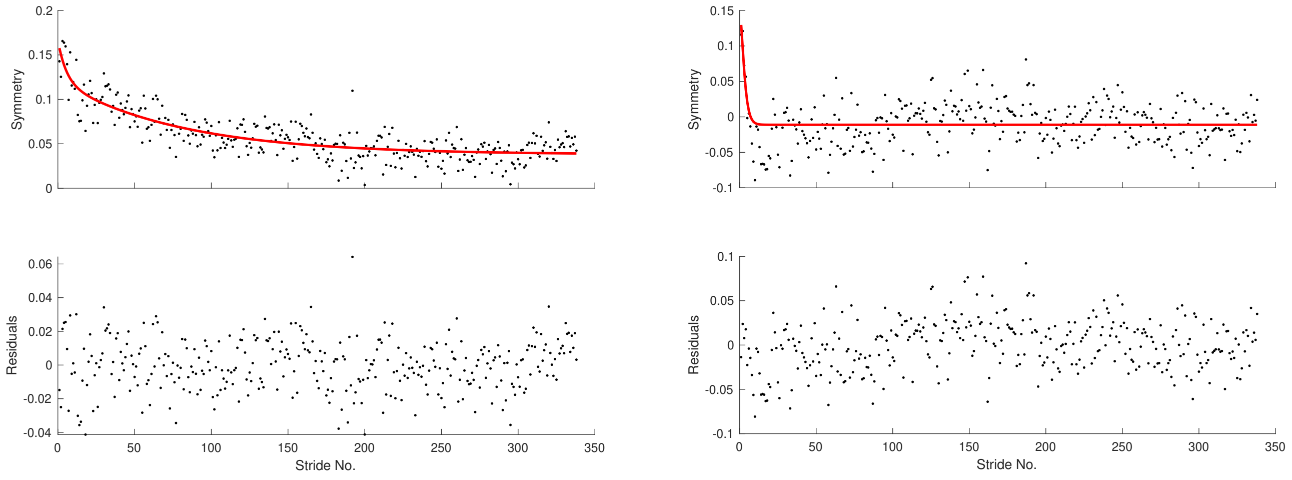

Participant 11, 12

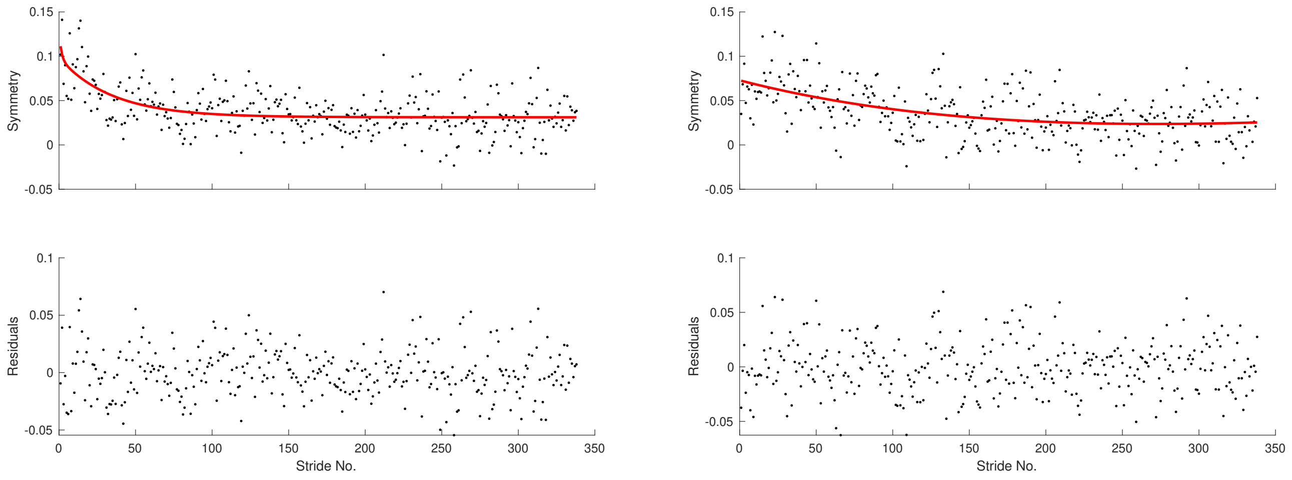

Participant 13, 14

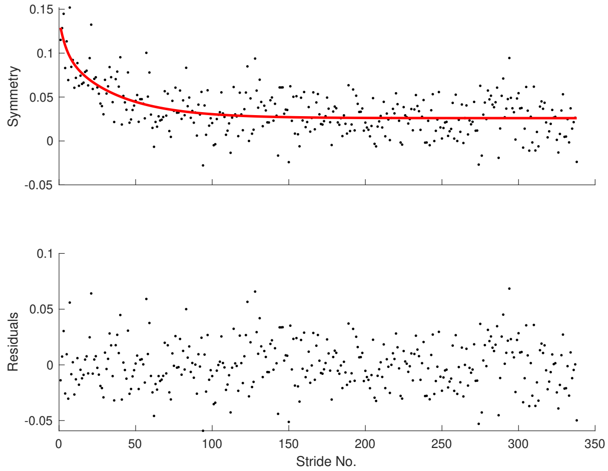

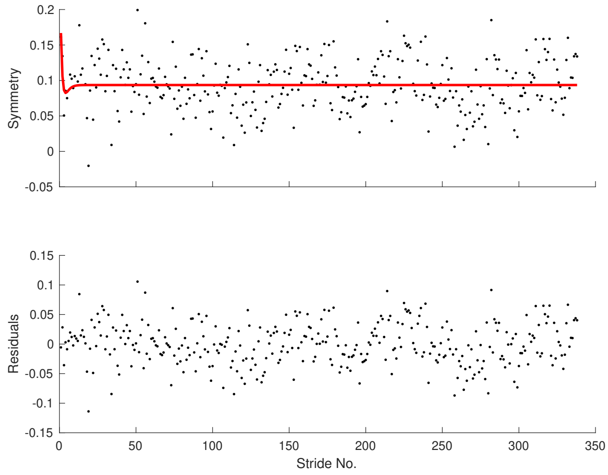

Participant 15

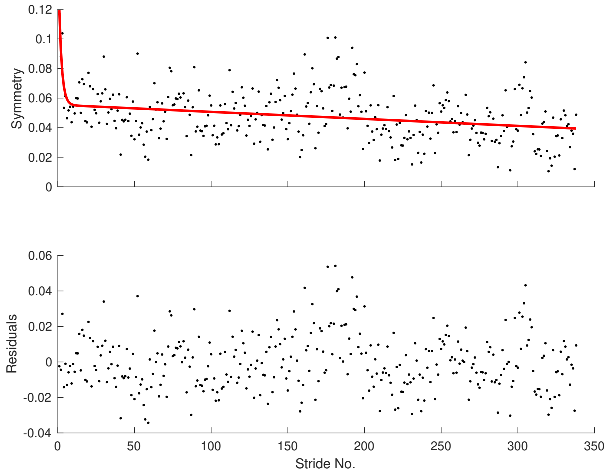

Supplement: Supplementary file 1 [file brainsci-10-00737-s001.pdf]
